# Atomistic Mechanism of Lipid Membrane Binding for Blood Coagulation Factor VIII with Molecular Dynamics Simulations on a Microsecond Time Scale

**DOI:** 10.1021/acs.jpcb.4c06575

**Published:** 2025-01-22

**Authors:** Nathan G. Avery, Kenneth C. Childers, James McCarty, Paul Clinton Spiegel

**Affiliations:** Chemistry Department, Western Washington University, Bellingham, Washington 98225-9038, United States

## Abstract

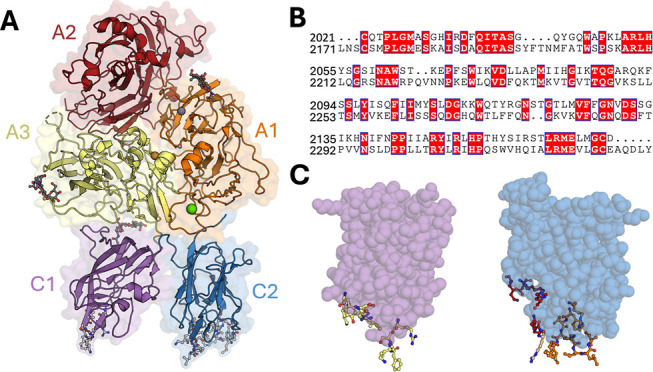

During the blood coagulation cascade, coagulation factor VIII (FVIII) is activated by thrombin to form activated factor VIII (FVIIIa). FVIIIa associates with platelet surfaces at the site of vascular damage to form an intrinsic tenase complex with activated factor IX. A working model for FVIII membrane binding involves the association of positively charged FVIII residues with negatively charged lipid headgroups and the burial of hydrophobic residues into the membrane interior. Currently, the atomic details of the FVIII lipid binding interactions and membrane orientation are lacking. This study reports residue-specific FVIII C domain interactions with 1,2-dioleoyl-*sn*-glycero-3-phosphocholine (DOPC) and 1,2-dioleoyl-*sn*-glycero-3-phospho-l-serine (DOPS) in atomistic detail. Contact maps between residues in the C domains with different lipid moieties support prior structural data describing how the C domains associate with membranes through electrostatic and hydrophobic interactions. Solvent-accessible surface area analysis quantified the extent to which residues in the C1 and C2 domains bury into the membrane. Calculations of the potential energy between the C domains and DOPC and DOPS revealed an FVIII membrane-binding orientation that agrees with previous experimental data. This study expands our knowledge of the structural basis of FVIII membrane association, which may be critical for the development of next-generation FVIII replacement constructs with improved activity.

## Introduction

Vascular damage initiates the blood coagulation cascade to repair and maintain hemostasis.^1,2^ Coagulation factor VIII (FVIII) is an essential protein in the intrinsic pathway of the blood coagulation cascade and circulates in the blood while bound to its carrier protein von Willebrand factor (vWF) to block premature uptake by dendritic cells and proteolysis.^3−6^ Once the cascade is initiated, FVIII is activated by thrombin to form activated FVIII (FVIIIa), causing dissociation from vWF^7^ and binding to activated platelets presenting phosphatidylserine (PS) and phosphatidylethanolamine (PE) on the outer membrane leaflet. FVIIIa acts as a cofactor for activated factor IXa (FIXa), forming the intrinsic tenase complex, which catalyzes the cleavage of factor X (FX) to form activated factor X (FXa) and amplify thrombin production.^3,7,8^ Disruption of FVIII expression and/or function due to genetic mutation causes congenital hemophilia A, an X-linked bleeding disorder that affects 1 in 5000 male births.^9^ Of these, missense mutations can affect various FVIII procoagulant functions, such as binding to vWF, FIXa, and activated platelet surfaces.^10^

**Figure 1:**
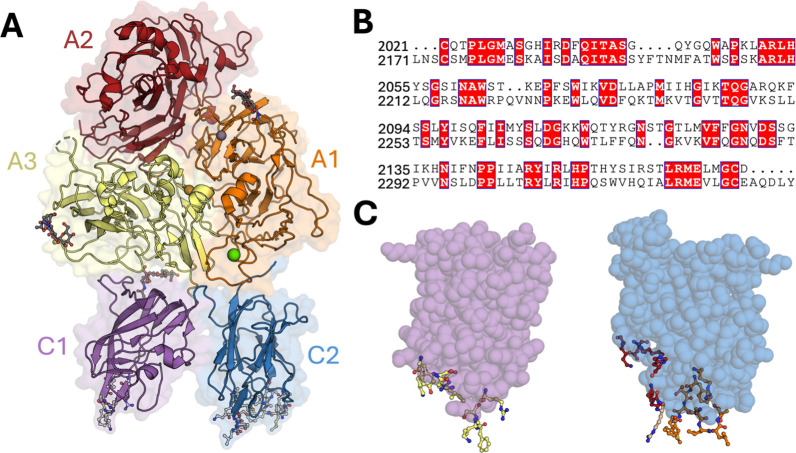
FVIII Domain Organization and Antibody Epitopes. (A) Blood coagulation factor VIII domain organization A1 (orange), A2 (red), A3 (yellow), C1 (purple), and C2 (blue) PDB ID: 6MF2. Metals are colored as follows: zinc (gray), copper (orange), and calcium (green). N-linked glycans are shown as sticks and colored dark gray. FVIII C domain β-hairpin loops are shown as sticks in light gray. (B) Sequence alignment of the C1 domain (residues 2021-2170) and C2 domain (residues 2171-2332) made using the ENDScript Server.^25^ Conserved residues are colored white. (C) Antibody epitopes of C1 (purple) and C2 (blue) domains (PDB ID: 6MF2). Residues involved in the KM33 antibody epitope are shown as yellow sticks. Residues involved in the BO2C11 (orange sticks) and 3 × 10^6^ (red sticks) or both (brown sticks) antibody epitopes are shown.

FVIII is a 2332 amino acid residue glycoprotein that is expressed predominantly in liver sinusoidal endothelial cells with a domain architecture consisting of A1-A2-B-A3-C1-C2 (Figure 1A).^6,11−13^ Prior to secretion into circulation, the B domain is differentially cleaved, and FVIII is processed and trafficked as a heterodimer consisting of a heavy chain (A1-A2-B) (residues 1-1313) and a light chain (A3-C1-C2) (residues 1648-2332). The B domain is not required for procoagulant function and is removed in several recombinant FVIII replacement products, so-called B domain-deleted (BDD) FVIII protein constructs.^13^ Upon vascular damage, FVIII is activated through proteolytic cleavage by thrombin at residues R372, R740, and R1689 to form FVIIIa, yielding a heterotrimeric assembly consisting of A1/A2/A3-C1-C2.^6,11,13^ Following activation, FVIIIa associates with activated platelet membranes predominantly through the C-terminal domains C1 (residues 2021–2170) and C2 (residues 2171–2332).^11,14,15^ The C1 and C2 domains are discoidin-like domains that display a β-barrel sandwich fold with 40% sequence identity (Figure 1B) between each other and bind to PS-containing lipid membranes with high affinity.^10^

A working model for the FVIIIa membrane binding that agrees with the majority of prior structural and biochemical data consists of both C domains associating with activated platelet surfaces via electrostatic and hydrophobic interactions through a coplanar interdomain arrangement.^11,15−18^ Crystal structures of FVIII have revealed the locations of hydrophobic (F2093, M2199, F2200, L2251, and L2252) and positively charged (K2092, R2159, R2163, R2215, R2220, K2249, and R2320) amino acids on the C domains that favorably interact with lipid membranes.^11,19^ Some of these residues (K2092, F2093, M2199, F2200, K2249, L2251, and L2252) are located as β-hairpin loops in the C domains shown as sticks in Figure 1A. Epitope analysis of pathogenic antibodies that inhibit FVIII cofactor function and site-directed mutagenesis has also given insight into FVIII membrane binding. Crystal structures of the isolated C2 domain bound to inhibitors 3E6 and BO2C11, which potently block FVIII binding to lipid membranes, revealed key residues predicted to be involved in membrane association. The 3E6 epitope consists of C2 domain residues K2183, D2187, R2209, and Q2213-R2215. The BO2C11 epitope consists of C2 domain residues S2250-T2253, F2196-F2200, R2215, R2220, Q2222-V2223, and H2315-Q2316 (Figure 1C).^16,17,20^ The recent structure of BDD FVIII bound to KM33, an anti-C1 domain inhibitor, highlights a patch of positively charged residues on the C1 domain (R2090, K2092, and R2159) that are also key for FVIII membrane binding.^21^ The KM33 epitope is C1 domain residues Y2043, Q2045, K2065, K2092-S2094, and I2158-R2159 (Figure 1C).^21^

Studies incorporating alanine substitutions to the FVIII C domains with membrane-binding measurements have expanded our understanding of residues involved in lipid interactions.^15,22,23^ When C1 domain residues R2090/Q2091, K2092/F2093, R2159, or Q2042/Y2043 were mutated to alanine, FVIIIa displayed reduced cofactor activity as well as binding affinity for PS-containing membranes.^15^ Similarly, when C2 domain residues M2199/F2200 and L2251/L2252 were mutated to alanine, FVIII possessed decreased binding affinity for PS membranes.^22^ These structural and biochemical studies demonstrate that these residues are important determinants of FVIII cofactor activity and membrane binding. The structure of FVIII C2 domain bound to a small-molecule membrane-binding inhibitor (PDB ID: 3HNB) revealed residues W2313, V2314, and H2315 are involved in FVIII C2 domain membrane binding.^24^ Leveraging molecular dynamics (MD) to study time-resolved FVIII-lipid interactions can yield insight into the FVIII membrane-binding orientation, analysis of protein–lipid interaction energetics, and specificity of residues for different lipid groups.

This study advances our investigations into the structural and energetic basis for how FVIII associates with PS-containing lipid membranes. We performed MD simulations of the isolated C1 domain, isolated C2 domain, and BDD FVIII with an 80% 1,2-dioleoyl-*sn*-glycero-3-phosphocholine (DOPC) and 20% 1,2-dioleoyl-*sn*-glycero-3-phospho-l-serine (DOPS) lipid nanodisc. These simulations are consistent with prior experimental data and support the interaction between the FVIII C domains and lipid membranes through both hydrophobic and electrostatic interactions. Previously reported work by others^26−31^ employed MD simulations to study the C domains of the C domains of FVIII, or homologous proteins FV and lactadherin, and revealed the discoidin-like C domains interact with PS-containing membranes via electrostatic and hydrophobic interactions. In our work, we present longer simulations, up to 1 μs of BDD FVIII, the isolated FVIII C1 domain, and the isolated FVIII C2 domain at atomic resolution in the presence of a lipid nanodisc model that emulates an activated platelet outer membrane leaflet. This system is of interest because it reports a comprehensive simulation of BDD FVIII with model lipid membranes, which is directly comparable to prior experimental work.^32^ We quantified the observed interactions by measuring contacts between residues in the C1 and C2 domains with different regions of the DOPC and DOPS over time. Root mean square fluctuation (RMSF) analyses revealed that membrane-buried residues in the C domains are stabilized by the presence of the A domains in BDD FVIII simulations. Calculation of the solvent-accessible surface area (SASA) per residue allowed us to quantify the extent of membrane burial per residue. Finally, measurement of the energetics between the C domains and DOPC and DOPS revealed an FVIII membrane-binding orientation on a lipid nanodisc.

## Materials and Methods

### MD Simulations

Activation of platelets stimulated by thrombin and collagen contain 12–15% PS, 40% phosphatidylcholine, 28% PE, and 20% sphingomyelin.^33,34^ To simplify the lipid system used and closely align it to prior FVIII membrane-binding experiments,^32,35−38^ an 80% DOPC and 20% DOPS composition was chosen for the model membrane. The 80% DOPC/20% DOPS nanodisc was prepared using CHARMM-GUI^39−42^ with the CHARMM36m all-atom force field.^43^ The assembled nanodisc had a diameter of 97 Å and a height of 47 Å and contained 64 DOPC and 16 DOPS molecules per leaflet for a total lipid surface area of 5606 Å. Isolated FVIII C1 (residues 2021–2170) and C2 (residues 2171–2332) domains were prepared from a recent X-ray crystal structure of BDD FVIII (PDB ID: 6MF2).^19^ Full length BDD FVIII was prepared from the full-length human FVIII AlphaFold by the deletion of residues in the B domain (residues 743–1636) to produce a BDD FVIII model (residues 1–742 and 1637–2332) that incorporates loops that are unmodeled in existing X-ray structures (AlphaFold ID: AF-P00451-F1).^44,45^ The AlphaFold structure quality was assessed by alignment with the human FVIII crystal structure with an RMSD of 2.04 Å. The isolated C1 and C2 domains were oriented approximately 3–6 Å above the membrane surface to ensure that the C domains were proximal to the nanodisc during the simulation. For BDD FVIII/nanodisc simulations, the C domains were oriented approximately 8 Å above the membrane surface to ensure the membrane proximity. Simulation details are provided in Table S1. Pronation states of residues and lipid molecules used GROMACS 2019 default settings of canonical p*K*_a_ values of residues and a pH value of 7.^46^ Missing termini of each protein construct were added and not capped. Each system was equilibrated with GROMACS 2019^46^ before converting to Desmond dms format for Anton2. The system was solvated with TIP3P water and additional sodium and chloride ions to neutralize it and achieve a salt concentration of 0.15 M. A steepest descent energy minimization was performed until the maximum force on any atom was less than 1000 kJ/mol/nm. The system was then equilibrated at 300 K for 100 ps using the stochastic velocity rescaling thermostat^47^ with position restraints on all heavy protein atoms.^47^ The system was then equilibrated in the *NPT* ensemble at 1 bar and 300 K for 6 ns without restraints using the Parrinello–Rahman barostat.^48^ Bonds to hydrogen atoms were constrained using the LINCS algorithm.^49^ The resulting equilibrated structures were used to run two independent 1 μs simulations at 310 K and 1 bar using the Anton2 machine maintained by the Pittsburgh Supercomputing Center (PSC) and made available by D.E. Shaw Research (DESRES).^50^ Atomic coordinates were saved every 240 ps.

### Analysis of MD Simulations

The number of contacts between two groups of atoms was calculated using the PLUMED 2.4 plugin^51,52^ to measure contacts between C1, C2, and C1C2 domains with DOPS and DOPC. A contact is considered formed if the distance between the groups is less than 4 Å. The number of contacts is the sum of all contacts between atoms in the indexed C1 or C2 group and the lipid group that have a distance closer than 4 Å, where the sum is over all elements in the C1 or C2 and lipid groups. The sum is one if the distance between two atoms in the C1 or C2 groups is less than 4 Å and zero if it is greater than 4 Å. Atoms were indexed for each lipid group as follows: carbons of the DOPC or DOPS lipid tail (carbon chain DOPS or DOPC), carbons of the DOPC or DOPS headgroup (choline carbon DOPC or serine carbon DOPS), ester oxygens of DOPC or DOPS (ester O DOPC or DOPS), nitrogens on DOPC or DOPS (N DOPS or DOPC), phosphate of DOPC or DOPS (PO_4_ DOPC or DOPS), or carboxyl group of the headgroup of DOPS (carboxyl DOPS) (Figure 3A). For the C domain residues, nitrogen and oxygen atoms were indexed for all residues, and carbon atoms were indexed for all hydrophobic residues.^51,52^ Hydrophilic contacts were measured between nitrogen and oxygen atoms, indexed together per C domain residue, and the following lipid groups: ester O DOPC and DOPS, N DOPS and DOPC, PO_4_ DOPC and DOPS, and carboxyl DOPS. Hydrophobic contacts were measured between carbon atoms on hydrophobic C domain residues and the following groups: carbon chain DOPS and DOPC, choline carbon DOPC, and serine carbon DOPS.

**Figure 2:**
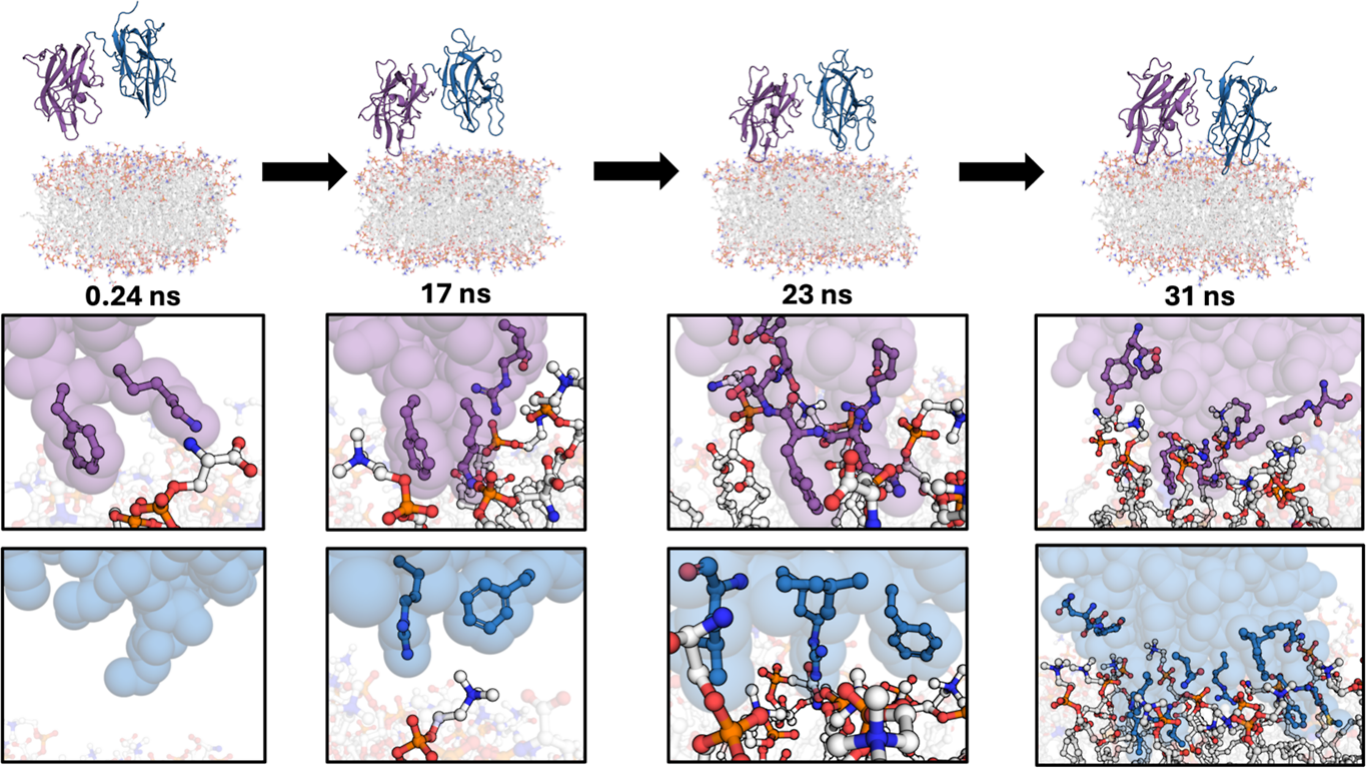
Lipid Binding Progression of C Domains of BDD FVIII to a Lipid Nanodisc. The C domains are colored: C1 domain (purple) and C2 domain (blue). During the BDD FVIII simulation, the C1 domain bound to the nanodisc first followed by the C2 domain. Interacting residues (less than 4 Å) in the C domains with DOPS and DOPC are shown as sticks. The FVIII A domains and nanodisc scaffold protein are not shown, and lipids are colored in white.

**Figure 3:**
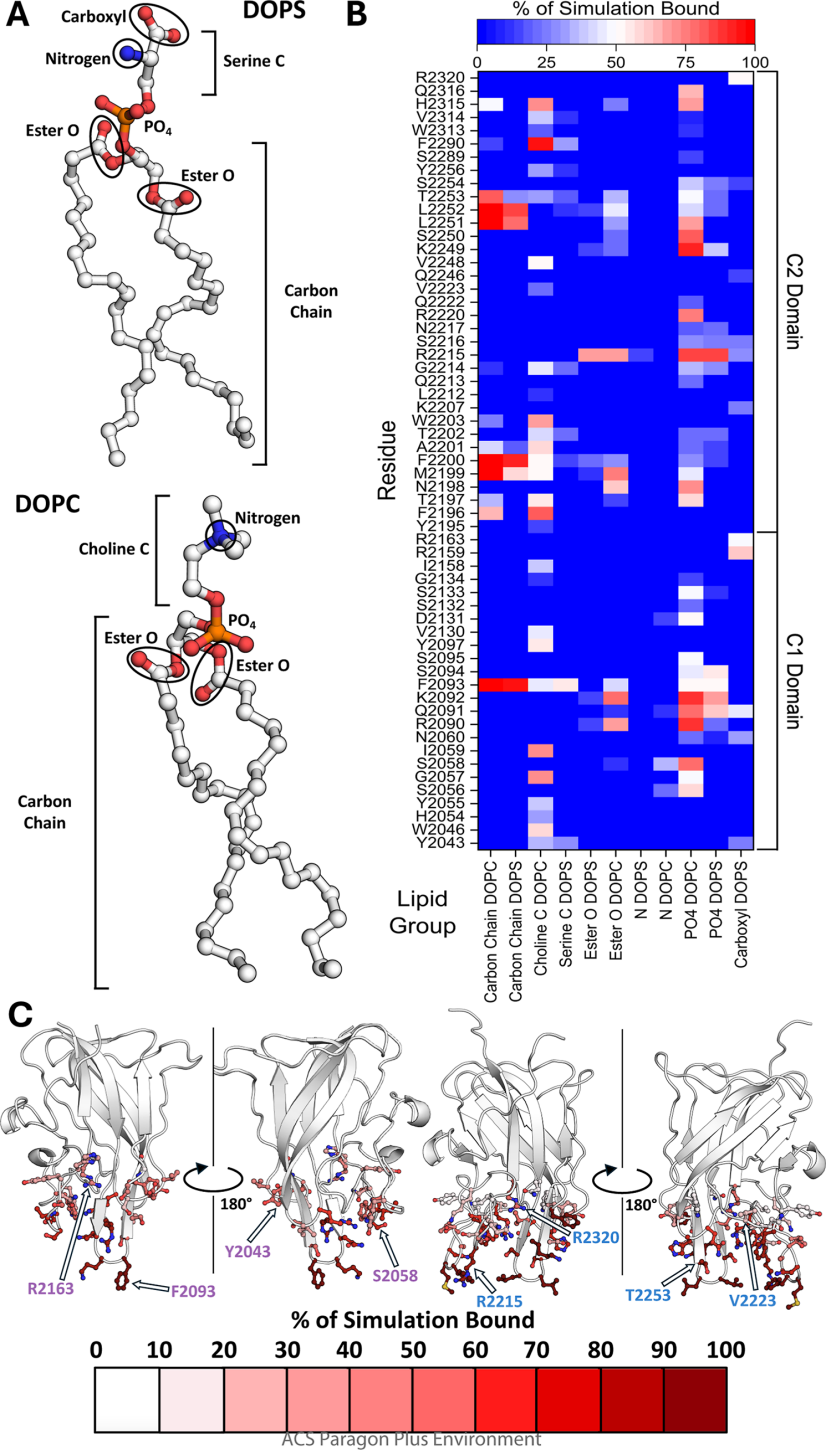
Contacts between FVIII C domains and DOPS and DOPC. (A) DOPS and DOPC Lipid Groups for Contact Map. Atoms are colored as follows: blue (nitrogen), red (oxygen), orange (phosphorus), and white (carbon). (B) Heat map of contacts between BDD FVIII C1 and C2 domains with different lipid groups. A cutoff distance of 4 Å was used to determine whether a contact is formed. Data are representative of the average of two trials. (C) Contact Map of the BDD FVIII C1 Domain (left) and C2 Domain (right) with a Lipid Nanodisc (PDB ID: 6MF2). Contacting residues are depicted with ball-and-stick representation. The nanodisc is not shown. Data are representative of the average of two trials.

RMSF of the backbone atoms per residue, root-mean-square deviation (RMSD) of backbone atoms, and SASA^53^ per residue for C1, C2, and C1C2 domains were all calculated with GROMACS^46^ analysis tools. Potential energy calculations were also performed with GROMACS^46^ between C1, C2, and C1C2 domains and DOPC and DOPS.^46^

The radial distribution functions (RDF) for the binding of R2163 (C1 domain) and R2320 (C2 domain) to the oxygen atoms in the DOPS carboxylate functional group were calculated using GROMACS. RDF values were normalized in GROMACS according to the relative positions of the reference group (R2163/R2320 guanidino nitrogens), the volume of the bin, and the average particle density of the positions of the selected group (oxygens of the DOPS carboxylate functional group).^46^ The RDF for residues R2163 and R2320 and the carboxylate functional group of DOPS were calculated as they have been previously shown to be required for FVIII-PS membrane association.^32^ Previous studies have calculated the potential mean force (PMF) to measure the free energy profile of protein–lipid interactions.^54−56^ The PMF was calculated from the radial distribution function (*g*(*r*)) at 310 K as PMF = −*RT* ln[*g*(*r*)]. PMF was calculated from RDF as a starting point to estimate the effective free energy profile of the R2163/R2320-DOPS interaction due to the RDF measurement of the probability of finding particles at a given interparticle distance. This provides a quantitative measure of the local order around R2163/R2320. RDF was used to estimate the most probable distance of R2163/R2320 from the nanodisc surface.

The lowest potential energy frame of the BDD FVIII trajectory (Figure S1) was aligned with FVa in the FVa/FXa bound to a nanodisc cryo-EM structure (PDB ID: 7TPQ)^57^ in PyMol. FIXa was prepared from the human full-length FIX AlphaFold model by removal of the processing (residues 1–46) and activation peptides (residues 192–227) (AlphaFold ID: AF-P00740-F1)^44,45^ and was aligned with FXa in the FVa/FXa structure in PyMol.

The tilt angles of the C1, C2, and A domains relative to the nanodisc membrane were calculated using the PLUMED 2.4 plugin,^51,52^ as shown in Figure S2. The alpha carbons of three residues around the scaffold protein of the nanodisc, M32 (point 1), E93 (point 2), and E158 (point 3), were chosen to represent the membrane surface. A vector was calculated between points 1 and 2 and 1 and 3. Then, the cross product of vector12 and vector13 was computed and normalized by  where . A protein vector was calculated between the alpha carbons of residues K510 and G2136 (A domains), I2145 and F2093 (C1 domain), and P2299 and F2200 (C2 domain), as shown in Figure S2. The tilt angle was calculated by subtracting 90° minus the dot product, , between the membrane normalized vector and protein vector over the course of the 1 μs simulations.

## Results

### Simulations of FVIII with a Nanodisc Illustrate the C Domain Lipid Binding Mechanism

Throughout the duplicate, 1 μs MD simulations of BDD FVIII with a lipid nanodisc, the C1 domain bound to the membrane first, followed by the C2 domain once the C1 domain had initiated burial of its β-hairpin loop (Figure S3). This study observed primarily a two-step membrane-binding process where the hydrophobic β-hairpin loop of the C1 domain embedded into the membrane, allowing the hydrophobic loops of C2 to get closer to the membrane for insertion. This is followed by the C domain adoption of a tilt angle averaging 68° ± 8° (C1 domain) and 69° ± 9° (C2 domain) (Table S2, Figures S4 and S5) once all β-hairpin loops were initially anchored to the membrane (Figure 2).

### C1 and C2 Domains of FVIII have Differential Contacts with Lipid Nanodiscs

Contacts between FVIII C domains and lipid groups in the nanodisc were measured over the duplicate 1 μs simulations (isolated C1 domain, isolated C2 domain, and BDD FVIII) to analyze how FVIII interacts with a PS-containing lipid membrane over time. A contact map was generated for the isolated C1 domain (Figure S6A), C2 domain (Figure S6B), and BDD FVIII with the lipid nanodisc (Figure 3), and contacts were tabulated between FVIII C1 and C2 domain residues in all simulations with various lipid moieties. For approximately 50% of the BDD FVIII simulations, conserved FVIII amino acids R2163 and R2320 were observed (Figure S7)^10^ and were bound to the carboxylate functional group of DOPS (Figure 3). By contrast, for the isolated C1 domain and isolated C2 domain simulations, R2163 (C1 domain) was bound for 16% (Figure S6A) and R2320 (C2 domain) for 60% (Figure S6B) of the simulations to the carboxyl group on DOPS, respectively. In BDD FVIII simulations, surface-exposed hydrophobic residues F2093, M2199-F2200, and L2251-L2252 were in contact with DOPS and/or DOPC for 98–99% of the simulation. Additionally, polar and charged basic residues in the C1 domain (S2058, R2090, Q2091, K2092, and R2159) and C2 domain (N2198, R2215, R2220, K2249, S2250, H2315, and Q2316) made contacts with polar groups on DOPS and/or DOPC for greater than 50% of the simulation. Overall, the C2 domain has more surface-exposed hydrophobic residues (and therefore more surface area) that make contacts with the lipid carbon chain in comparison to the C1 domain. The C1 domain only has one residue, F2093, that makes contacts with the carbon chain of DOPS and/or DOPC for a longer duration of the simulation (>50% of time). Residues of the C2 domain that made carbon–carbon contacts for greater than 50% of the simulation with DOPS and/or DOPC are F2196-T2197, M2199-A2201, L2251-T2253, and H2315. These results suggest that the C1 domain may harness a membrane-binding mechanism different from that of the C2 domain, despite the sequence homology (Figure 1B) between the two domains.

Contacts were monitored between residues in the BDD FVIII C domains with DOPC and DOPS lipids in a model lipid nanodisc that mimics activated platelet membranes.^33,34^ Lipid contacts that persisted for the greatest amount of time (>90% of the simulation) were on the C domain β-hairpin loops, as described above. FVIII C1 domain membrane binding differs from the C2 domain, as the C1 domain has only one β-hairpin loop that buries into the membrane for >90% of the simulation, whereas the C2 domain harbors two. In the isolated simulations, the C1 and C2 domains were also oriented at a different angle compared to the BDD FVIII simulations. The C1 domain in the isolated C1 domain-nanodisc simulations adopted a tilt angle of 31 ± 16° (Figure S8 and Table S2) compared to 68 ± 8° in the BDD FVIII-nanodisc simulations (Figure S4 and Table S2). The C2 domain in the isolated C2 domain-nanodisc simulations adopted a wide distribution of tilt angles centered at 10 ± 52° (Figure S9 and Table S2) compared to the 69 ± 9° in the BDD FVIII-nanodisc simulations (Figure S5 and Table S2). This difference in orientation of the C domains could partially explain why the isolated C domains have μM affinity for PS membranes, while BDD FVIII has nM affinity for PS membranes.^32^ Another possible explanation for FVIII increase in PS membrane-binding affinity could be because FVIII contains three arginine (R2159, R2163, and R2320) residues that interact with the carboxylate functional group of DOPS (Figure 3B) compared to the isolated domains having only one arginine (C2 domain, Figure S6B) or two arginines (C1 domain, Figure S6A) that interact with the carboxylate functional group of DOPS. Indeed, the C1 domain in BDD FVIII is oriented with R2163 facing toward the membrane (Figure S4), while the C2 domain displays a similar orientation in the BDD FVIII simulation with a nanodisc, where R2320 is also oriented toward the membrane surface (Figure S5). This reorientation correlates with other residues surrounding R2163 and R2320 having increased binding with the membrane surface, which are listed in Figures 3B and S10.

### Backbone Fluctuation of Isolated C1 and C2 is Significantly Higher than C1 and C2 in BDD FVIII

**Figure 4:**
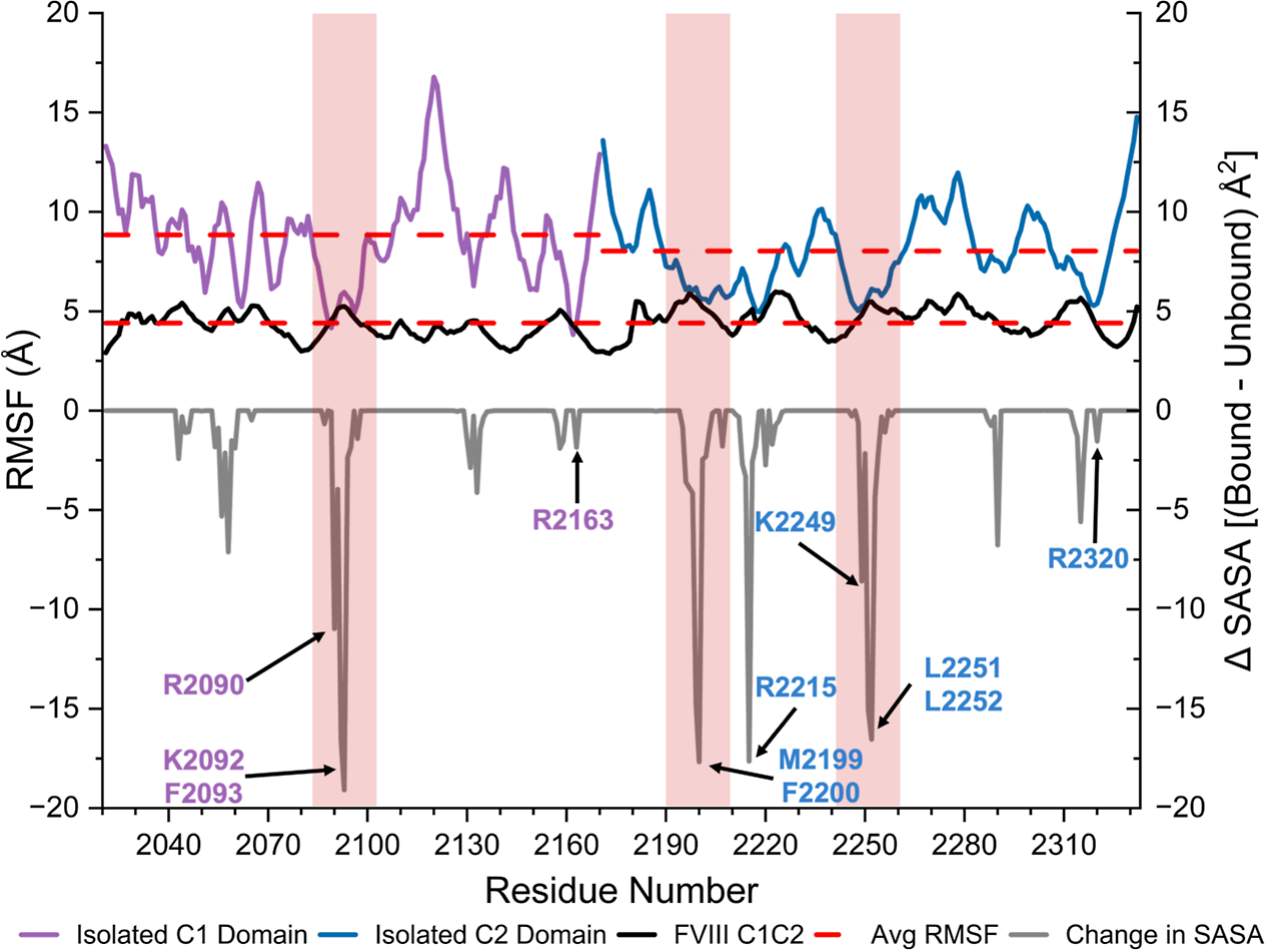
Backbone RMSF for Isolated C1, Isolated C2, and C1C2 Domains in BDD FVIII and Change in SASA per Residue upon Membrane Binding for BDD FVIII C Domains. Isolated C1 domain (purple), isolated C2 domain (blue), and BDD FVIII C1 and C2 domains (black) backbone RMSF for simulations with a nanodisc. Dashed red lines indicate the average backbone RMSF for the isolated C1 domain, isolated C2 domain, or BDD FVIII C1 and C2 domains. The change in SASA upon lipid membrane binding (ΔSASA) was calculated by subtracting the average SASA of the bound C domain from the unbound C domain per residue throughout the 1 μs simulation. C1 domain residues are highlighted in purple, and C2 domain residues are highlighted in blue. β-hairpin loop residues are highlighted in red. Data are representative of the average of two trials.

The RMSF values for the C1 and C2 domains were calculated for isolated C domain and BDD FVIII simulations to analyze how the presence of the A domains affected the fluctuation of C domain residues during membrane binding. The average RMSF per residue for each C domain was twofold lower in the BDD FVIII simulation compared to those for the isolated C1 and C2 domains (Figure 4). These results highlight the putative contribution of the A domains to the overall stabilization of the interrelated orientations for the tandem C domains. Despite this difference in the average RMSF, membrane-buried residues K2092–F2093, M2199-F2000, and L2251-L2252 had similar RMSF for all MD simulations with BDD FVIII and isolated C1/C2 domains (Figures 4 and S11), suggesting that these residues are constrained by interactions with the lipid membrane.

### Several Residues are Buried in the Membrane Interior upon C Domain-Nanodisc Binding

The SASA values for each residue were calculated for BDD FVIII in the presence and absence of a nanodisc to determine the extent of membrane burial (Figure 4). Residues with the most dramatic change in SASA reside on the C domain β-hairpin loops; specifically, residues R2090, K2092–F2093, M2199-F2200, R2215, K2249, and L2251-L2252 had decreases of 10 Å^2^ or greater in SASA upon membrane binding. Residues R2163 and R2320 had a smaller change in SASA compared to the membrane buried residues of about 2 Å^2^, likely because these two residues are largely buried in the protein core apart from their respective guanidino functional groups (in the undocked BDD FVIII structure (PDB ID 6MF2), R2163 has 8.5 Å^2^ SASA and R2320 has 8.0 Å^2^ SASA, exclusively due to their guanidino functional groups). Residues S2056 and S2058 in the C1 domain and H2315 and F2290 in the C2 domain had an average of a 6 Å^2^ change in SASA. These results indicate that the β-hairpin loops in the C1 and C2 domains underwent the most significant decrease in solvent accessibility upon membrane binding.

### Negatively Charged PS Binds to Positively Charged Regions on C1 and C2

**Figure 5:**
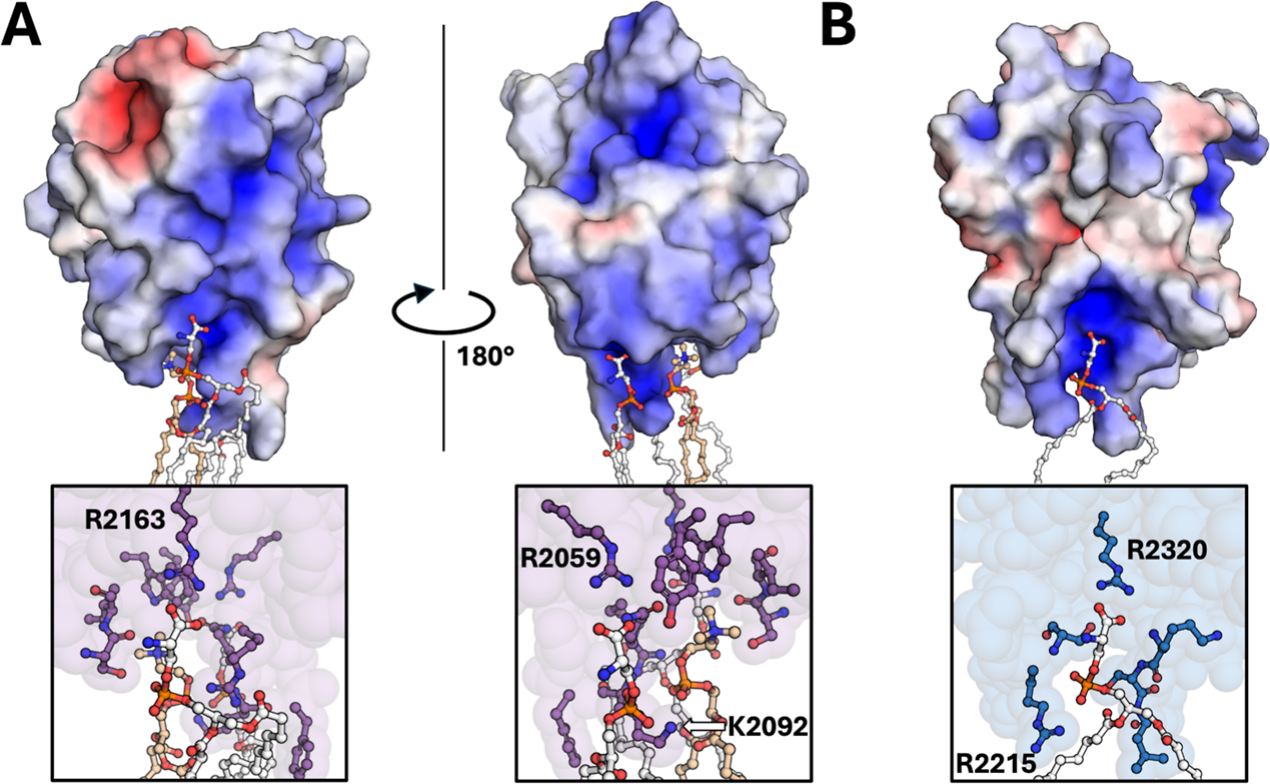
Lowest Energy Frame of Isolated C1 Domain R2163 and R2159 (A) and C2 Domain R2320 (B) Bound to DOPS. Hydrophilic residues R2163, R2159, and K2092 (A) and R2320 and R2215 (B) are labeled. DOPS is highlighted in white, and DOPC is highlighted in tan. Surface charge was calculated using the APBS plugin with a gain value of ±5 eV and visualized in PyMol.

The electrostatic surface in the lowest potential energy frame is C1 (Figure S12) and C2 (Figure S13) bound to PS was cataloged for each of the isolated C1 and C2 domain simulations (Figure 5). The carboxylate group of DOPS centers its binding interaction directly with the guanidino groups of R2163 (C1 domain) and R2320 (C2 domain), which agrees with prior simulation data of the isolated C domains and a PS-containing membrane.^26^ Another DOPS carboxylate group contact is on the opposite side of the C1 domain with the guanidino group of R2159 (Figure 5A). Residue K2092 in the C1 domain also makes additional electrostatic interactions with the phosphate groups of DOPS and DOPC. The C2 domain residue R2215 makes a similar contact with the phosphate of DOPS, which is the same DOPS molecule to which R2320 makes direct contact. This interaction was identified in the lowest potential energy frame of the isolated C2 domain simulation (Figure 5B and S13). Both electrostatic maps indicate that the hydrophobic residues in the C1 and C2 domains interact mostly with the hydrophobic interior of the phospholipid membrane. Electrostatic and hydrophobic interactions appear to collectively stabilize DOPS in a binding orientation to the C1 and C2 domains. These structural findings are consistent with previous experimental work indicating that R2163 and R2320 are critical for PS membrane association.^32^

### FVIII Simulations with Lipid Nanodiscs Reveal a Putative FVIIIa Binding Orientation for the Intrinsic Tenase Complex

**Figure 6:**
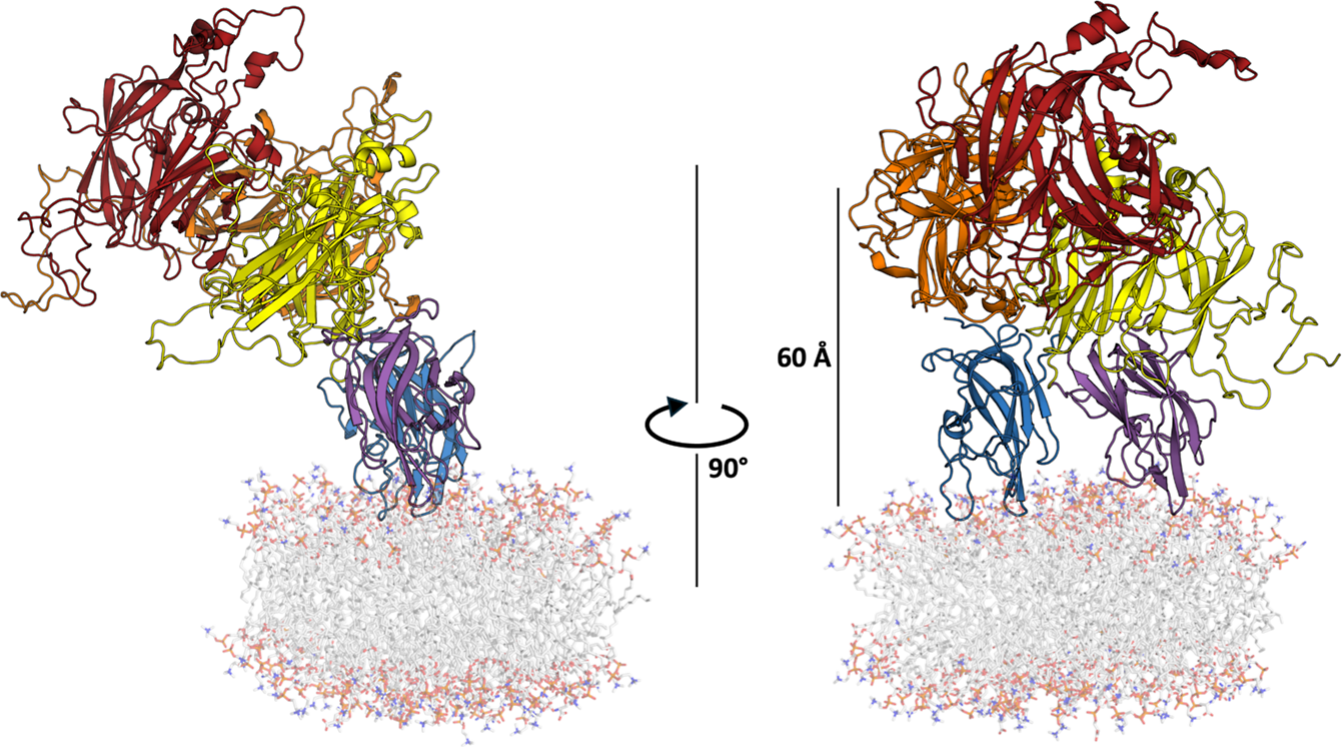
Lowest Energy Frame of BDD FVIII Bound to a Lipid Nanodisc Reveals Possible FVIIIa Binding Orientation for FIXa Binding. BDD FVIII domains are colored as follows: A1 (orange), A2 (red), A3 (yellow), C1 (purple), and C2 (blue). The scaffold protein is not shown, and lipids are colored in white.

The lowest potential energy frame of the C1 and C2 domain contacts with the lipid nanodisc was calculated for BDD FVIII and nanodisc simulations (Figure 6 and S1), revealing a convergent FVIII binding orientation on membrane surfaces. In the lowest potential energy frame, the C domains adopted a stable conformation with a 69° (C1 domain) and 71° (C2 domain) tilt to the membrane, whereas the A domains are oriented 46° relative to the membrane surface. This orientation is similar to the FVIII membrane-binding orientation previously predicted, where the whole FVIII molecule adopts a 60° tilt relative to the membrane surface.^15^ Over the course of the 1 μs simulations, the A domains had more variance with a standard deviation of 13°, in the membrane binding tilt compared to the C domain standard deviation of 8° (C1 domain) and 9° (C2 domain) (Table S2 and Figure S14). It is possible this variance in the A domain tilt angle is to allow for FIXa to bind to the A2 domain proximal to the surface of activated platelet membranes. The lipid-binding orientation of FVIII C domains at a 70° angle and the A domains at a 46° angle from the membrane could orient FIXa in a favorable conformation and distance from the activated platelet membrane surface for efficient catalysis.

### Energetics and RMSD of FVIII C Domains Binding to a Lipid Nanodisc

The average potential energy, minimum PMF of R2163 (C1 domain) and R2320 (C2 domain) binding to DOPS, and average backbone RMSD for the C domains were calculated (Table 1). The energetics of C domain binding did not change significantly when the C domains were in isolation compared to BDD FVIII (Figures S12, 13, S15, and S16). The average energies were similar for both C domains at an average of approximately −18,000 kJ/mol. The minimum PMF of R2163 or R2320 binding to DOPS was also similar between simulations (−16 to −22 kJ/mol) (Figures S14–S17). These values are approximately the attractive free energy difference (favorable Δ*G*) between bound and unbound R2163/R2320. These numbers are a qualitative estimate of binding; however, they do demonstrate exergonic association of R2163/R2320 with DOPS. More well-established free energy methods such as umbrella sampling could be employed in future studies to determine the quantitative free energy of C domain-membrane association. Calculation of the RMSD over the 1 μs simulations showed that the presence of the A domains in BDD FVIII stabilized the relative orientations of the C domains. The average backbone RMSD decreased in C1 and C2 by 2.5-fold and 1.6-fold, respectively (Figures S21–S25). This stabilization is most likely due to the contacts that the C1 domain has with the A3 domain and the C2 domain with the A1 domain, as well as the C1–C2 interdomain contacts.

**Table 1:** Energetics and RMSD of C Domain Nanodisc Binding^a^

nanodisc simulation	average potential energy (kJ/mol)	minimum PMF (kJ/mol)	average backbone RMSD (Å)
isolated C1 domain	–18,200 ± 400	–16.00 at 2.68 Å	26.3 ± 2.6
isolated C2 domain	–18,300 ± 300	–18.94 at 2.70 Å	21.5 ± 2.5
BDD FVIII: C1 domain	–18,100 ± 400	–21.72 at 2.72 Å	10.4 ± 1.0
BDD FVIII: C2 domain	–18,400 ± 400	–21.83 at 2.72 Å	13.3 ± 1.2
BDD FVIII: C1C2 domains	–19,200 ± 400		12.0 ± 1.1

a Energies and backbone RMSD were calculated in GROMACS. PMF between the guanidino group of R2163 (C1 domain) and R2320 (C2 domain) with the carboxyl group of DOPS was calculated in GROMACS. Data are representative of the average of two trials with a 95% confidence interval

## Discussion

### FVIII C Domains Associate with PS Containing Membranes via Electrostatic and Hydrophobic Interactions

Our results indicate that the C1 domain binds to the membrane first, allowing for the C2 domain to bind to the membrane (Figures 2 and S3). Previous experimental and simulation data have suggested that the C1 domain cooperates with the C2 domain for membrane binding.^15,58^ However, the order in which the C domains bind to the membranes is unclear. A previous coarse-grained in silico study of the C domains and a PS membrane found that the C2 domain bound to the membrane before the C1 domain,^58^ inconsistent with this study. These conflicting results could be due to the use of coarse-grained rather than all-atom simulations or because the C domains have no preference for which binds to the membrane first. It is possible that the coarse-grained simulations are missing atomic details that are important for the C domain’s membrane-binding process. Simulation of the C1 and C2 domains together without the A domains present allows for flexibility in orientation between the C domains that may influence the C domain membrane-binding order. Our simulations not only represent all atoms in the membrane and BDD FVIII but also simulate a complete FVIII protein assembly instead of the isolated C1 and C2 domains together, which allows for a more accurate representation of the FVIII membrane-binding process. Despite this difference in binding order, the progression of C domain lipid binding was similar. Du et al. also proposed that there are three stages to C domain membrane binding.^58^ The first stage is the formation of long-range electrostatic interactions when the C domains are within 5 Å of the membrane, followed by anchoring the hydrophobic spikes (β hairpin loops containing residues F2093, K2092, M2199, F2200, L2251, and L2252) into the membrane, and the tilting angle stabilizes. The last step consists of the C1 and C2 domains reorienting into a stable conformation with an average tilt angle of 68 and 69° (Table S2), respectively, with optimized interactions at the membrane surface. The stages that the C domains bound in our simulations were consistent with this model, but instead of observing discrete steps, the C1 domain binds to the membrane surface centered on β-hairpin loops through both electrostatic and hydrophobic interactions initially, followed by C2 domain interaction predominantly through β-hairpin loop insertion (Figure 2). This difference could also be a consequence of our use of BDD FVIII in our simulations, which includes the stabilizing impact of the A domains.

**Figure 7:**
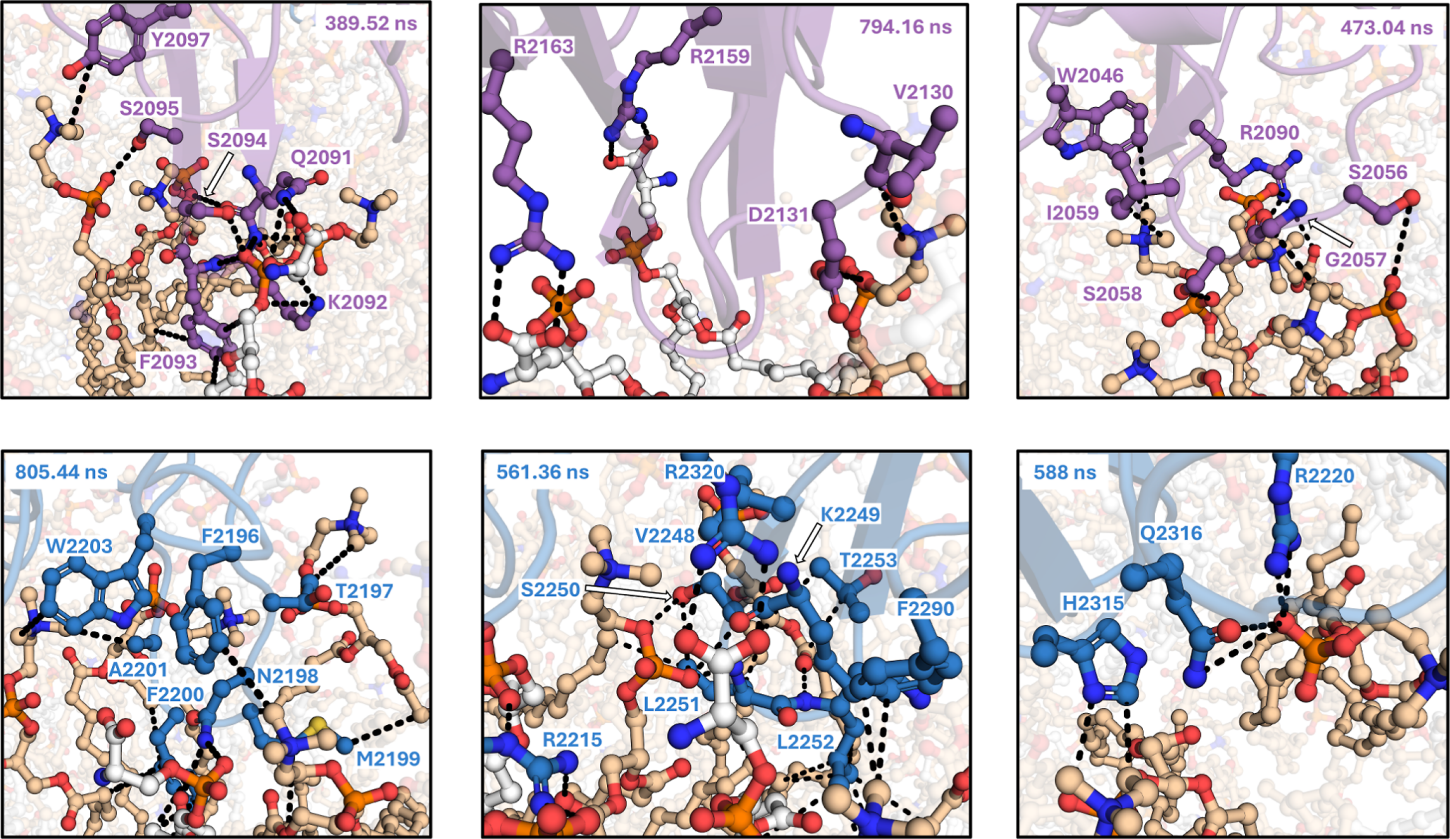
Snapshots of residues in the C domains that make contacts with DOPS and DOPC for greater than 50% of the BDD FVIII-nanodisc simulation. The C1 domain is colored in purple, and the C2 domain is colored in blue. DOPC is shown in tan and DOPS in white. Hydrogens and the scaffold protein are not shown. Dashed lines indicate a contact between two atoms less than 4 Å.

Previous structural studies have proposed that the FV and FVIII C domains associate with activated platelet surfaces through both electrostatic and hydrophobic interactions.^17,18,59^ Analysis of the X-ray crystal structure of the isolated C2 domain and BDD FVIII revealed surface-exposed hydrophobic residues in β-hairpin loops on the C2 domain. The C1 domain contains one surface-exposed hydrophobic residue, F2093, while the C2 domain contains two adjacent loops with two surface-exposed hydrophobic residues each (M2199/F2200 and L2251/L2252).^18^ This study demonstrates that F2093 is the major residue in the C1 domain that associates with the carbon chains of DOPS and DOPC. The C1 domain residues W2046-G2057, I2059, and Y2097 made most of the total lipid hydrophobic contacts (greater than 50% of the 1 μs simulation) with DOPC solvent-exposed methyl groups. In contrast, the C2 domain has prominent hydrophobic contacts with the aforementioned β-hairpin loops and adjacent residues. Residues F2196, M2199, F2200, L2251, L2252, T2253, and H2315 were bound to the carbon tail of DOPS and/or DOPC for 50% of the simulation or greater (Figure 7). This agrees with previous data showing M2199, F2200, L2251, and L2252 are necessary for optimal FVIII activity.^22^ The C2 domain also had surface-level hydrophobic contacts with the methyl groups on the choline headgroup. Overall, the C2 domain has more contacts with the lipid membrane; however, many of these contacts lasted 20–40% of the simulation.

The C1 domain forms contacts with the phosphate groups of both DOPS and DOPC. Surprisingly, the major membrane-burying β-hairpin loop is composed of mostly hydrophilic residues. Of these residues, R2090, Q2091, K2092, S2094, and S2095 bind to the phosphate group of DOPC, and some of these residues (Q2091, K2092, F2093, and S2094) bind to the phosphate of DOPS for >50% of the simulation. The C2 domain also harbors several lasting phosphate contacts (greater than 50% of the simulation), including residues T2197-M2199, R2215, R2220, K2249-L2251, T2253, and H2315-Q2316, which bind to the DOPC phosphate group for over half of the simulation (Figure 7). In contrast to the C1 domain, the C2 domain has fewer residues that directly bind to the phosphate group of DOPS for more than 50% of the simulation, with R2215 being the primary interaction. Previous studies have hypothesized that R2215 stabilizes the same phosphate moiety that interacts with R2320,^10^ which was directly observed in our MD simulations. The C1 and C2 domains differ in the residues that associate with the phosphate backbone of DOPC and DOPS. The C1 domain mainly associates with hydrophilic residues, whereas the C2 domain associates via the backbones of hydrophobic residues and hydrophilic residues. This is likely due to the difference in hydrophilic versus hydrophobic residues in the β-hairpin loops between the two C domains, which is in agreement with a recent biophysical analysis of the C domain interactions with PS-containing lipid nanodiscs.^35^ In this study, the C1 domain membrane binding is enthalpically driven, while C2 domain binding is largely entropically driven.

In the BDD FVIII simulations, only two residues in the C1 domain (R2159 and R2163) and one residue in the C2 domain (R2320) were observed bound to the carboxylate group of DOPS for >50% of the simulation. This supports previous experimental evidence that R2163 and R2320 are essential for FVIII membrane association.^32^ Another homologous protein to the C2 domain, lactadherin C2 domain, was also found to bind to the carboxylate group of DOPS during MD studies.^29,60^ Mutational studies of FVIII have shown the importance of R2159 for FVIII activity; when mutated to alanine, FVIII clotting activity was 42% compared to wild type.^15^ Residues R2159, R2163, and R2320 are essential for FVIII membrane association because the negatively charged DOPS headgroup can form favorable electrostatic interactions with the positively charged clefts of the C1 and C2 domains seen in Figure 5. The interaction of arginine residues in the C1 and C2 domains with DOPS is likely crucial for FVIII stable membrane binding with specificity for PS-containing lipid membranes. These MD simulations agree with previous results on these conserved arginine residues^15,32^ and support a possible conserved PS binding motif between discoidin domains.

MD simulations supported the mechanism of FVIII antibody inhibitors KM33, 3E6, and BO2C11 that were previously shown to block FVIII membrane binding.^16,17,21^ During the BDD FVIII simulations, residues R2090, K2092-S2094, and R2159, which constitute a majority of the KM33 epitope (Figure 1C),^21^ bound to DOPS and/or DOPC for more than 50% of the simulation. Similarly, residues T2202-R2215 form a portion of the 3E6 epitope (Figure 1C) and were found to make direct contact with lipids.^16^ Similar results were also observed with FVIII residues F2196-R2200, S2250-T2253, and H2315-Q2316, which constitute the BO2C11 epitope.^17^ Analysis of membrane-interacting residues between the C domains and a nanodisc in our simulations provides more insight into how these antibody inhibitors block FVIII membrane association and how residues in these epitopes interact with a PS-containing membrane with the highest frequency. Comparing membrane-binding antibody inhibitor epitopes with the BDD FVIII simulation data supports the binding orientation that the C domains adopt in the 1 μs simulations.

### FVIII C Domain Residues R2163 and R2320 Shift Toward the Membrane Compared to Isolated C Domain/Nanodisc Simulations

When comparing the isolated C1 and C2 domain simulations (Figures S6A,B) to BDD FVIII simulations, R2163 bound to the headgroup of DOPS more frequently, approximately 17% (isolated C1 domain) in contrast to 50% (BDD FVIII C1 domain) (Figure 3B). When the C domains are adjacent in BDD FVIII, they are poised in a different orientation where R2163 and R2320 are oriented closer to the membrane. This can be observed when comparing Figure S10A,B, where contacts on the opposite side from R2163 and R2320 in the C1 and C2 domains decrease when the domains are in the context of BDD FVIII. It has been previously documented that the C domains in isolation have μM affinity for PS-containing membranes, but when in tandem, the C domains have nM affinity for the membrane.^32^ This change in orientation on the membrane, along with BDD FVIII containing two membrane-binding domains, explains the enhancement in binding affinity with BDD FVIII. The C1 and C2 domains have interdomain contacts that stabilize this conformation. Since residues R2163 and R2320 are necessary for FVIII high-affinity binding to PS membranes,^32^ it is possible that the orientation of these arginine residues in the C1 and C2 domains, along with other changes in electrostatic and hydrophobic interactions, contributes to the increase in binding affinity. This change in contacts is likely important for the stabilization of FVIII on the membrane surface for the formation of the intrinsic tenase complex.

### Analysis of FVIII-Nanodisc Simulations Yields a Putative Model for FIXa Binding to FVIIIa on a Membrane

**Figure 8:**
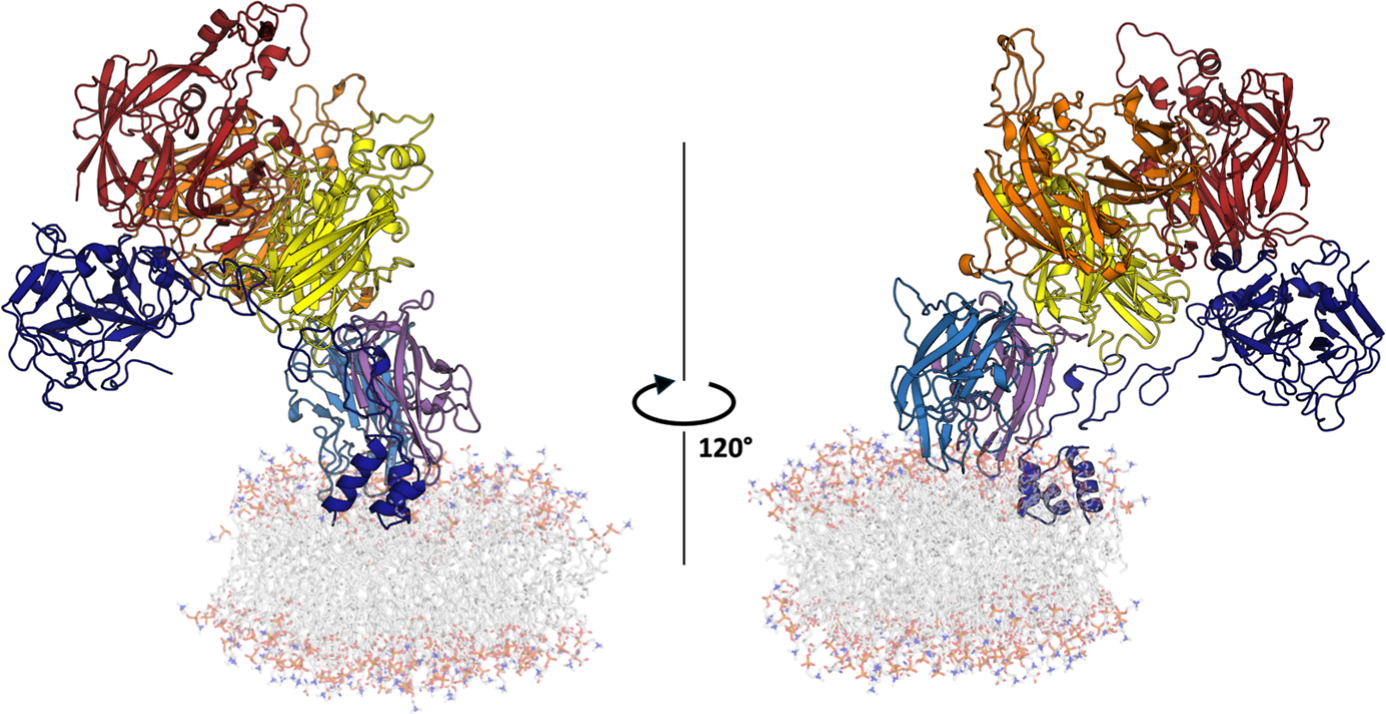
Modeling of the Tenase Complex on Lipid Nanodisc Based on the FVa/FXa Cryo-EM Structure. The lowest potential energy frame of FVIII bound to a nanodisc was aligned with the FVa/FXa cryo-EM structure. FIXa was then aligned with FXa from the FVa/FXa cryo-EM structure. The resulting alignment produced a possible tenase structure on the surface of a nanodisc. BDD FVIII domains are colored as follows: A1 (orange), A2 (red), A3 (yellow), C1 (purple), and C2 (blue). FIXa is represented in dark blue. The scaffold protein is not shown, and the lipids are colored in white.

The BDD FVIII structure from the lowest potential energy frame of the BDD FVIII simulation (Figure S1) was aligned with membrane-bound FVa, and FIXa was aligned with membrane-bound FXa from the FVa/FXa cryo-EM structure (PDB ID: 7TPQ).^57^ FV and FVIII share ∼40% amino acid sequence identity^61^ and structural homology (A1-A2-B-A3-C1-C2 domains)^11^ and have similar cofactor functions in the prothrombinase and tenase complexes, respectively.^62^ Previous studies have identified that FIXa interacts with the A2 domain of FVIII,^63^ and the 558 loop of the FVIIIa A2 domain is essential for FIXa activity.^64^ With similarities between the prothrombinase and tenase complexes, it is reasonable to hypothesize a similar binding motif. This model includes the protease domain of FIXa interacting with the A2 domain of FVIIIa, the FIXa EGF-1 and EGF-2 domains with FVIIIa C1 and A3 domains, respectively, and the FIXa Gla domain with the FVIIIa C1 domain (Figure 8). This alignment places the catalytic triad facing away from the A2 domain, where it can bind to FX. The positioning of the C domains with a 70° and A domains with a 46° tilt relative to the membrane surface is similar to the positioning of FVa in the prothrombinase complex.^57^ This could be a conserved orientation for the optimal FVIIIa and FVa cofactor activity to place FXa (prothrombinase) and FIXa (tenase) in optimal positions for proteolytic activation of prothrombin (prothrombinase) and FX (tenase), respectively. This model differs from other membrane-bound FVIII models, which have residues in the A1 and A3 domains^65^ or only the C2 domain interacting with the membrane surface.^66^

Previous studies incorporating low-resolution electron microscopy have proposed that the FVIII C domains undergo a drastic conformational change upon membrane binding, whereby the C1 domain is reoriented away from the membrane surface and the C2 domain adopts a side-lying conformation with the b-hairpin loops parallel to the membrane surface.^66^ Additionally, recent MD simulations incorporating the FVIII C1–C2 domains ligated together and a highly mobile membrane mimetic indicated that the C1 domain may adopt a similar side-lying conformation with the β-hairpin loops oriented outside of the lipid membrane.^29^ The MD simulations presented in this study revealed no significant conformational changes in either of the FVIII C domains in the context of the BDD FVIII protein. This observation is most likely due to the incorporation of the FVIII A domains, which appear to stabilize the relative conformations of the C1 and C2 domains; to adopt this reorientation of the C1 domain, it would require disruption of several hydrophobic interactions that represent a large, buried interface between the A3 and C1 domains.

## Conclusions

This study explores the membrane-binding orientation, energetics, and contacts between blood coagulation FVIII, a peripheral membrane-binding protein, and a model lipid nanodisc. These results are largely consistent with prior experimental data that have highlighted important residues in the C1 and C2 domains for the FVIII membrane association. The resultant contact map between FVIII and DOPC and DOPS has revealed for the first time in atomistic detail residues of interaction with specific groups on lipid molecules. The membrane-binding simulation for BDD FVIII demonstrates that membrane binding is initiated by the C1 domain, which serves to coordinate a subsequent stable C2 domain binding conformation. The structural details of membrane binding show that the C domains interact with lipids via both electrostatic and hydrophobic interactions to orient at a binding angle of approximately 60–70° in the most stable conformation. Direct comparison of the isolated C domain with full-length BDD FVIII simulations also provides a possible explanation for why FVIII affinity increases for PS-containing lipid membranes when the C domains are in tandem versus in isolation. Finally, a converged BDD FVIII lipid binding orientation was used to model the intrinsic tenase complex on a lipid nanodisc, yielding a putative orientation of FVIIIa that increases FIXa activity by 200,000-fold.

## Data Availability

All data are contained within this article and Supporting Information.

## Supporting Information Available

List of simulations run in this study, contact maps of isolated C domains and a lipid nanodisc, change in RMSF between BDD FVIII C domains and isolated C domains, backbone RMSD for isolated C domains and BDD FVIII C domains, potential energy between BDD FVIII C domains and isolated C domains and a nanodisc, radial distribution function and PMF of R2163 and R2320 interaction with the carboxyl group of DOPS, and contacts between C domains and a lipid nanodisc (PDF)
