# Supplementary material for: Atomistic Mechanism of Lipid Membrane Binding for Blood Coagulation Factor VIII with Molecular Dynamics Simulations on a Microsecond Time Scale
Source: J Phys Chem B. 2025 Jan 22;129(5):1486–98. doi: 10.1021/acs.jpcb.4c06575 (PMC11808648; doi:10.1021/acs.jpcb.4c06575)
Supplement: Supplementary file 1 — jp4c06575_si_001.pdf [file jp4c06575_si_001.pdf]

Supporting Information for

Atomistic Mechanism of Lipid Membrane Binding for Blood Coagulation Factor VIII with Molecular Dynamics Simulations on a Microsecond Timescale

Nathan G. Avery, Kenneth C. Childers, James McCarty, P. Clint Spiegel Jr.*

***Corresponding author:** P. Clint Spiegel, Jr., Chemistry Department, Western Washington University, 516 High Street, MS 9150, Bellingham, WA, USA; email: Paul.Spiegel@wwu.edu

Materials include:

Table S1 (pp. S2)

Figures S1-S19 (pp. S3-S22)

Supplemental Table

Table S1. List of simulations run in this study.

System with 80% DOPC 20% DOPS Nanodisc	Number of Replicates	Simulation Time (µs)	System Size (# of atoms)	Box Dimensions (nm)
Isolated C1 domain	2	1	231,859	13.1 x 13.1 x 13.1
Isolated C2 domain	2	1	231,703	13.1 x 13.1 x 13.1
BDD FVIII	2	1	845,943	20.2 x 20.2 x 20.2

Table S2. List of simulations run in this study. Data are representative of the average of two trials with standard deviation.

System with 80% DOPC 20% DOPS Nanodisc	Tilt Angle (Degrees)
Isolated C1 domain	31 ± 16
Isolated C2 domain	10 ± 52
BDD FVIII C1 domain	68 ± 8
BDD FVIII C2 domain	69 ± 9
BDD FVIII A domains	44 ± 13

Supplemental Figures

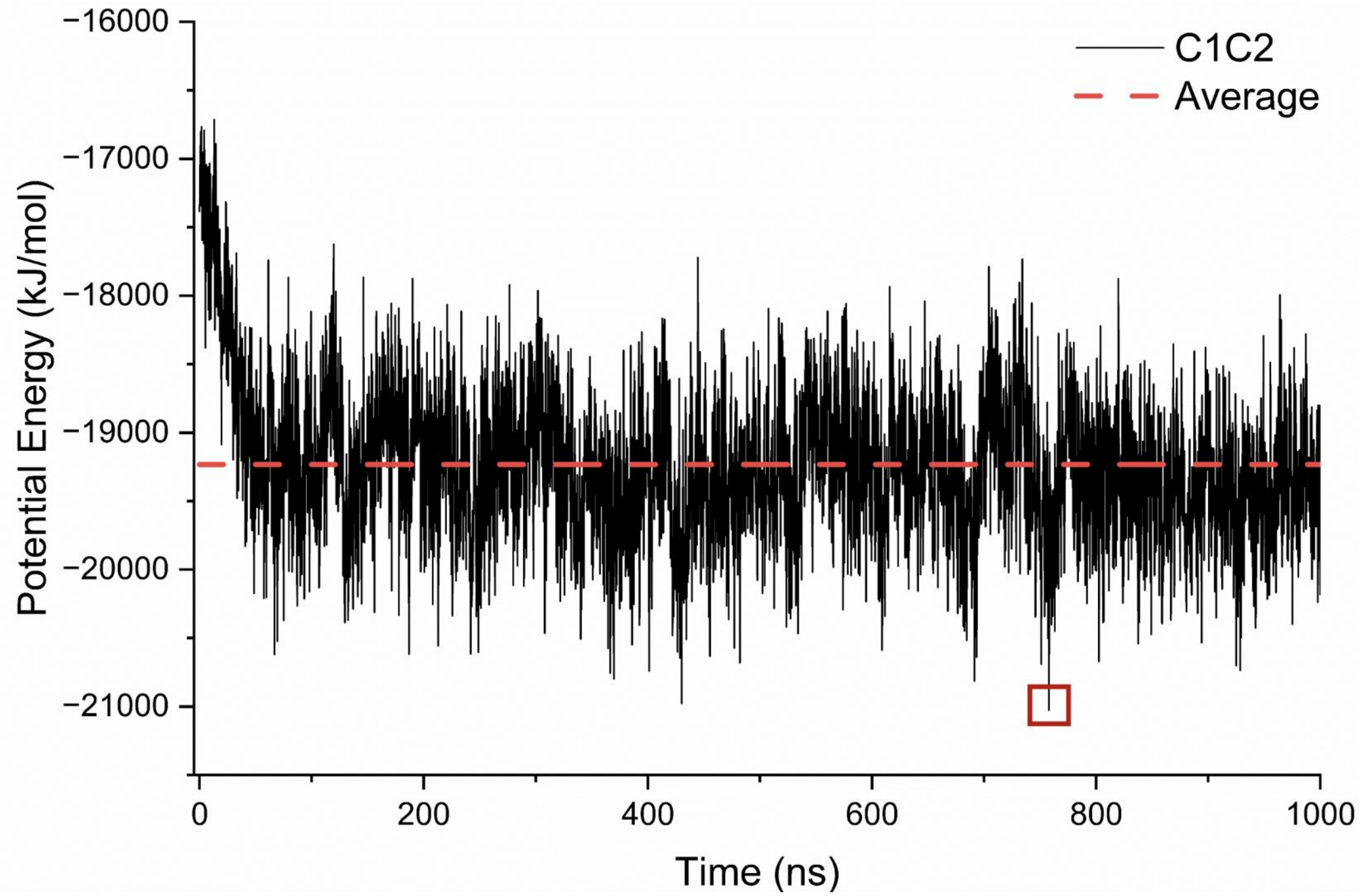

Figure S1. Potential energy between the C1 and C2 domain in the BDD FVIII, nanodisc simulations with DOPC and DOPS. Average potential energy is represented as a red dashed line. The lowest potential energy frame, 3158 (757.920 ns), is boxed in red. Data are representative of the average of two trials.

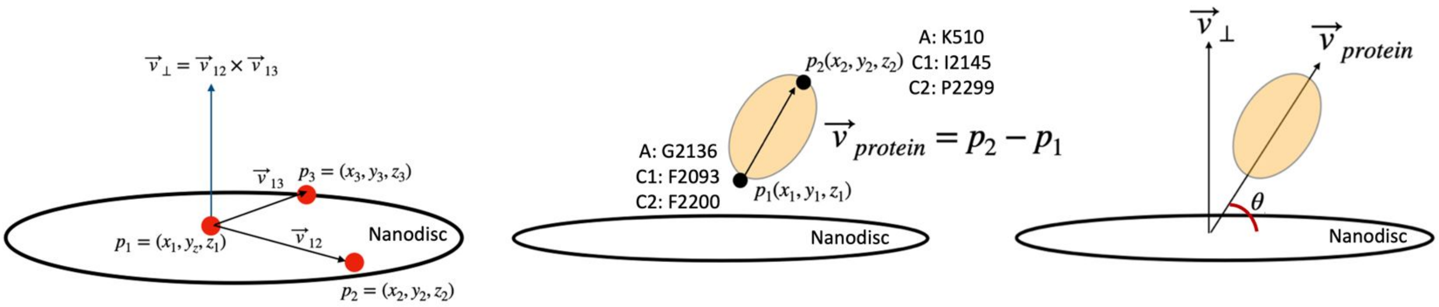

$$\hat{v} = \left(\frac{v_x}{|v|}, \frac{v_y}{|v|}, \frac{v_z}{|v|}\right)$$

$$|v| = \sqrt{v_x^2 + v_y^2 + v_z^2}$$

$$90° - \frac{\vec{v}_{\perp} \cdot \vec{v}_{protein}}{|v_{\perp}||v_{protein}|} = \cos(\theta)$$

Figure S2. Calculation of FVIII C domain tilt angle on nanodisc membrane.

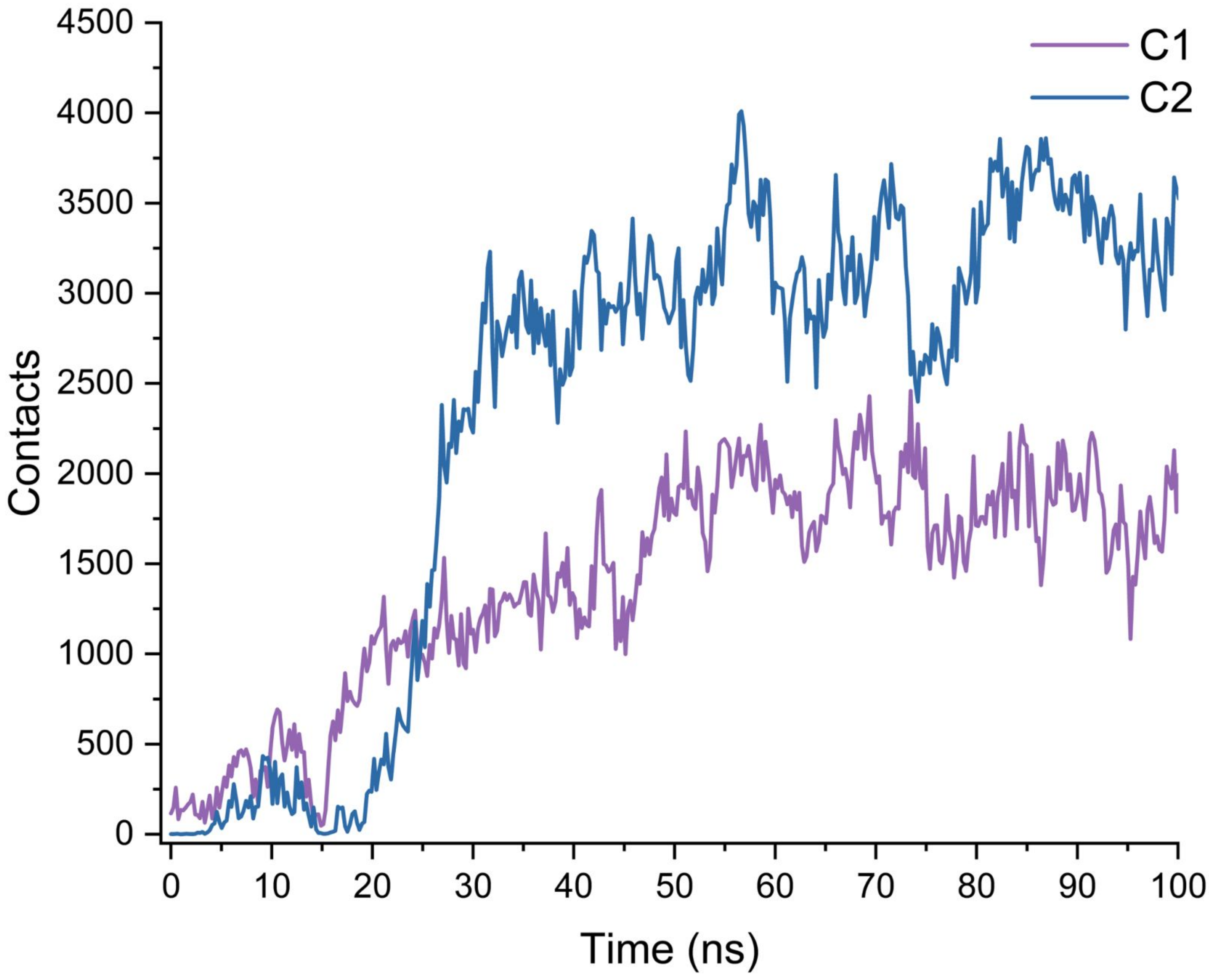

Figure S3. Contacts between C1 and C2 with a DOPC and DOPS in the lipid nanodisc. C1 contacts (purple) and C2 contacts (blue) are shown for the first 100 ns out of the 1 μs simulations. Data is representative of the average of two independent trials.

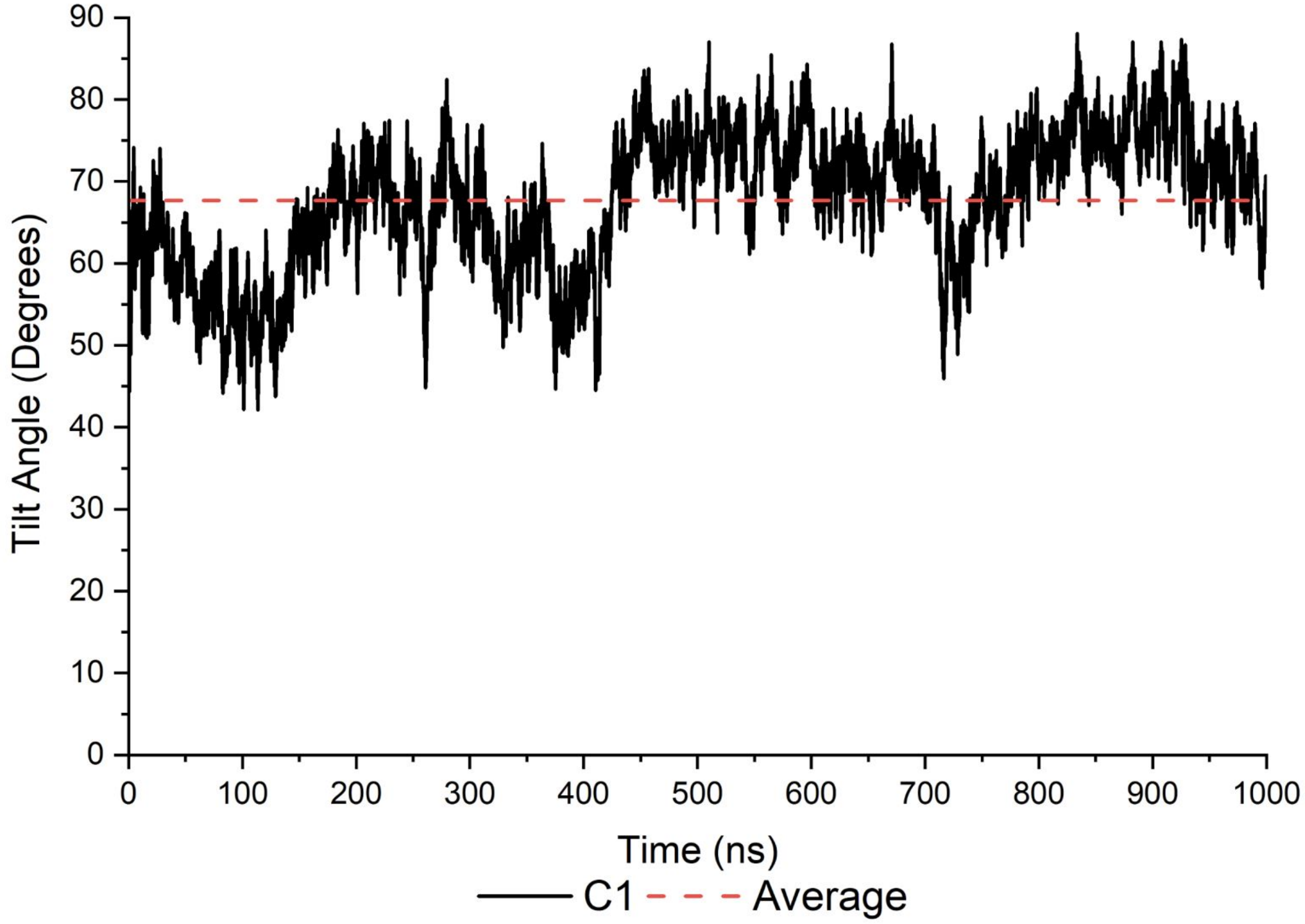

Figure S4. C1 domain tilt angle relative to the nanodisc membrane in the BDD FVIII-nanodisc simulations. The average tilt angle is represented as a red dashed line. Data are representative of the average of two trials.

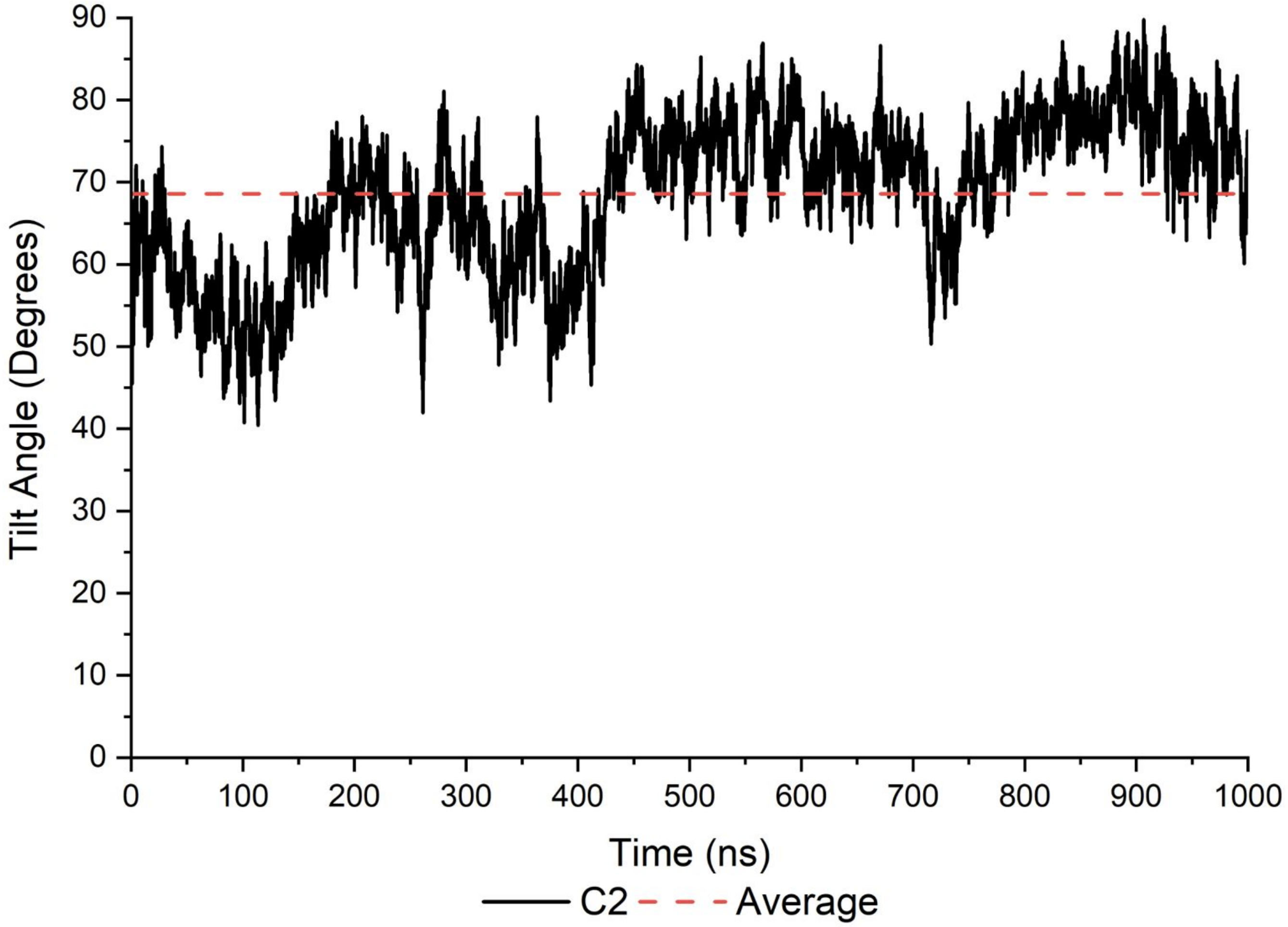

Figure S5. C2 domain tilt angle relative to the nanodisc membrane in the BDD FVIII-nanodisc simulations. The average tilt angle is represented as a red dashed line. Data are representative of the average of two trials.

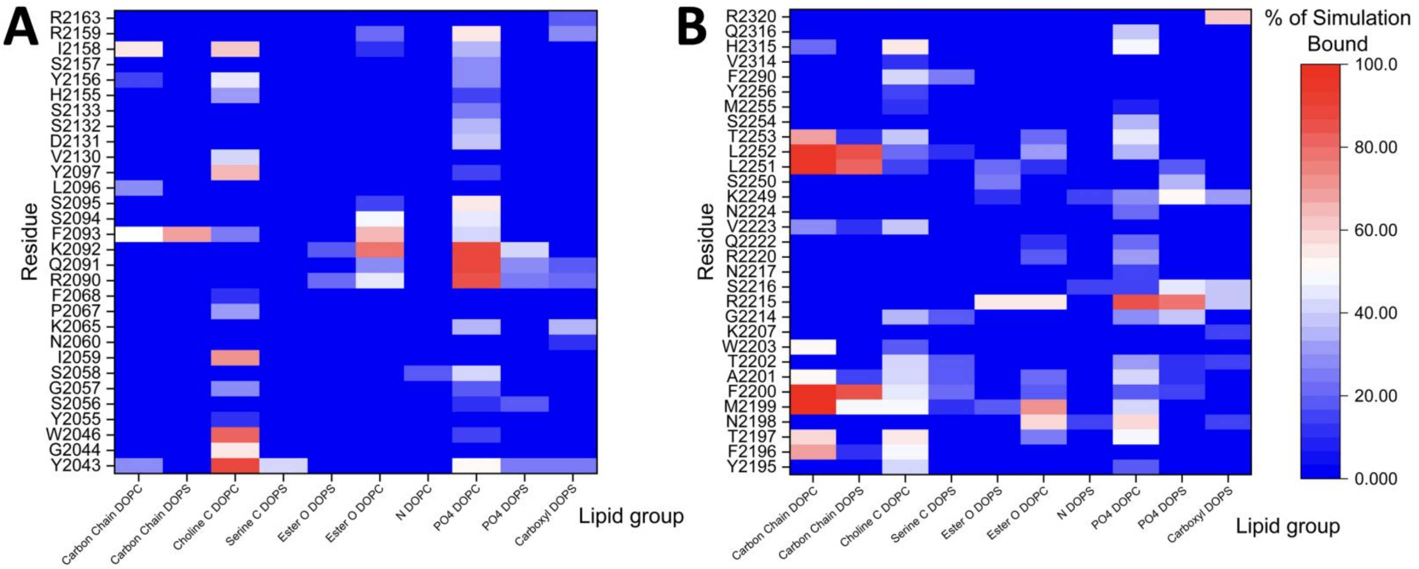

Figure S6. Heat map of contacts between isolated C1 (A) and C2 (B) domains with different lipid groups. A cutoff distance of 4 Å was used to determine whether a contact is formed. Data are representative of the average of two trials.

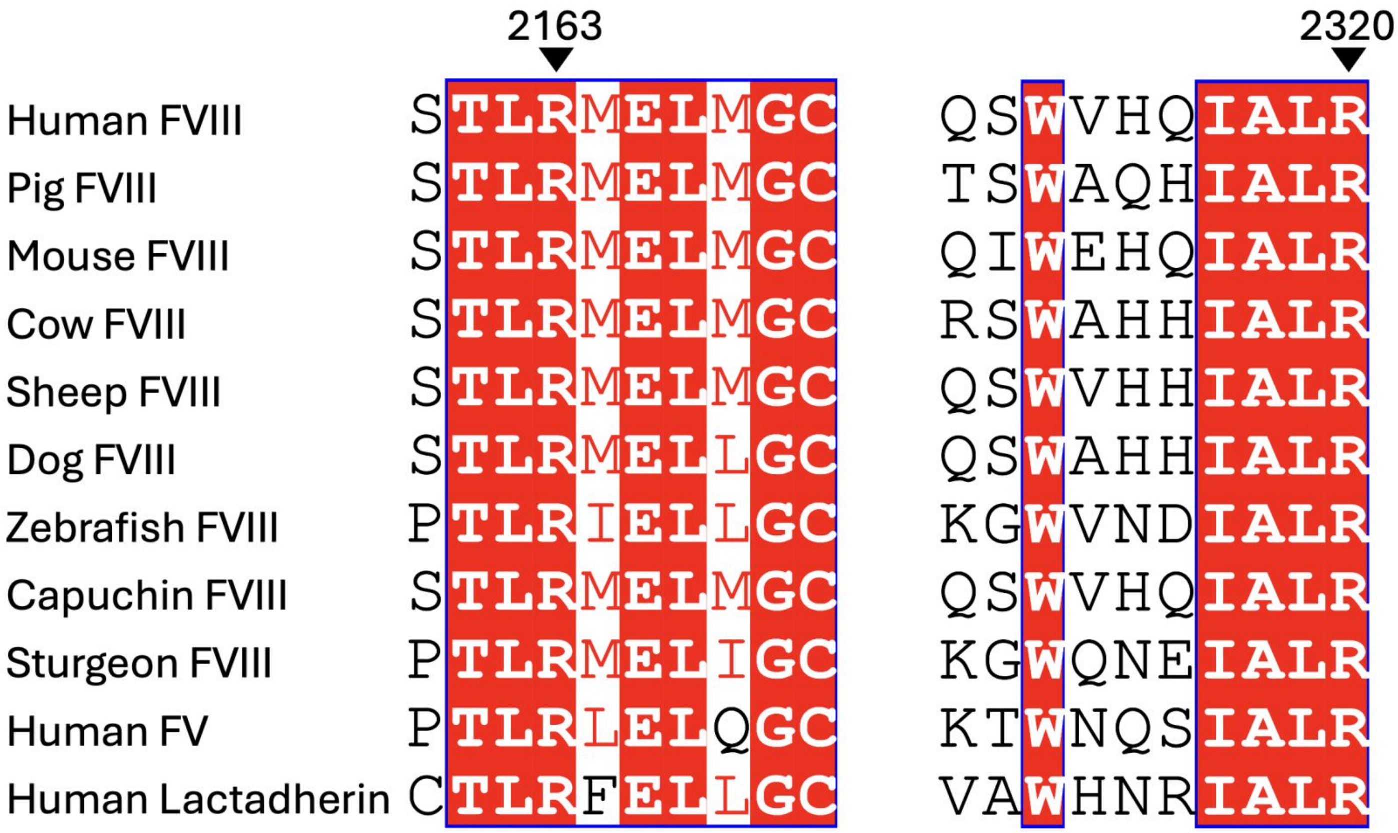

Figure S7. Multiple sequence alignment of various species FVIII, human FV, and human lactadherin. Conserved residues are colored white with red background.

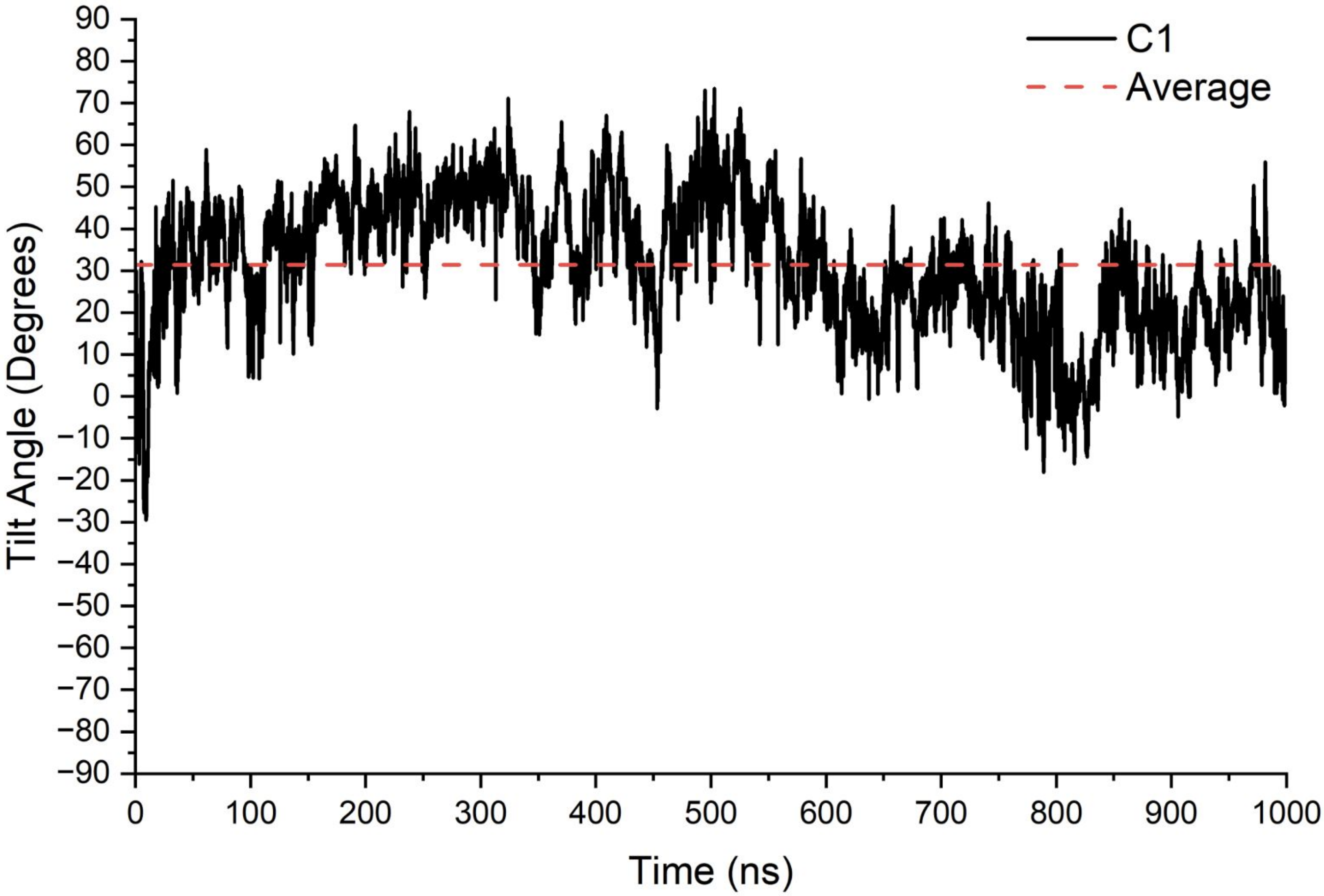

Figure S8. C1 domain tilt angle relative to the nanodisc membrane in the isolated C1-nanodisc simulations.

The average tilt angle is represented as a red dashed line. Data are representative of the average of two trials.

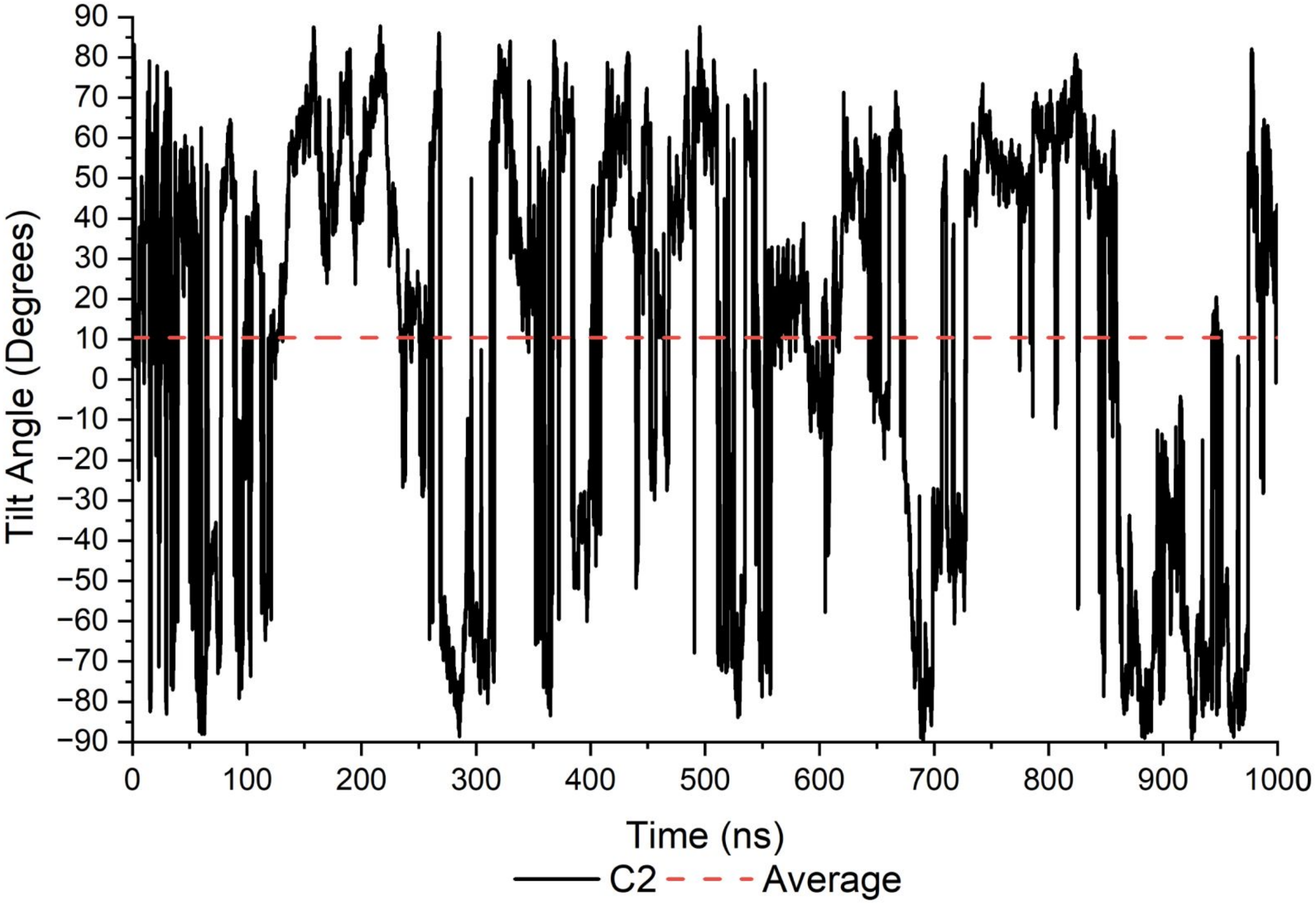

Figure S9. C2 domain tilt angle relative to the nanodisc membrane in the isolated C2-nanodisc simulations.

The average tilt angle is represented as a red dashed line. Data are representative of the average of two trials.

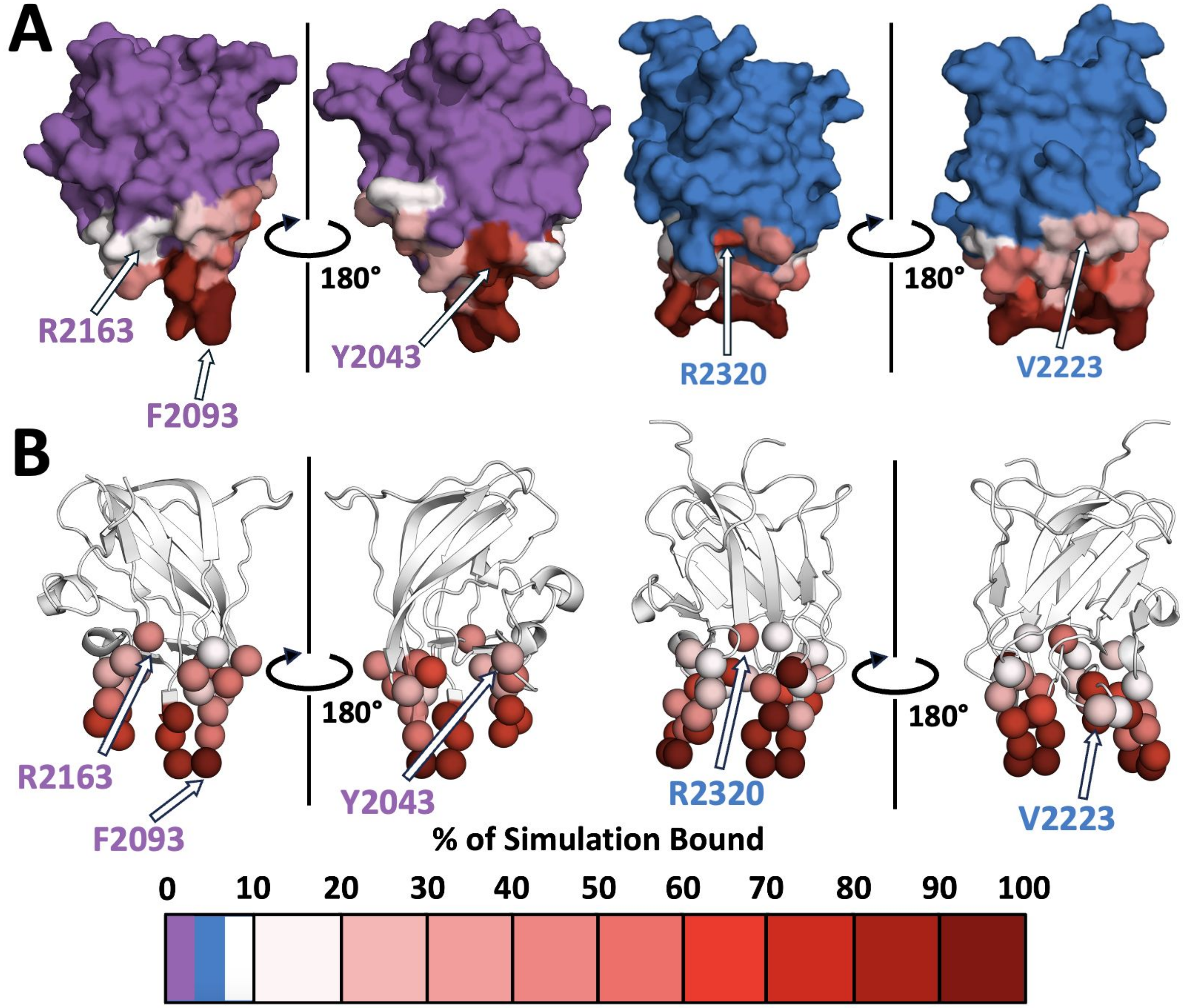

Figure S10. (A) Contact map of isolated C1 (purple) and isolated C2 (blue) with a lipid nanodisc. (B) Contact map of BDD FVIII C1 (left) C2 (right) domains with a lipid nanodisc. The nanodisc is not shown. Data are representative of the average of two trials.

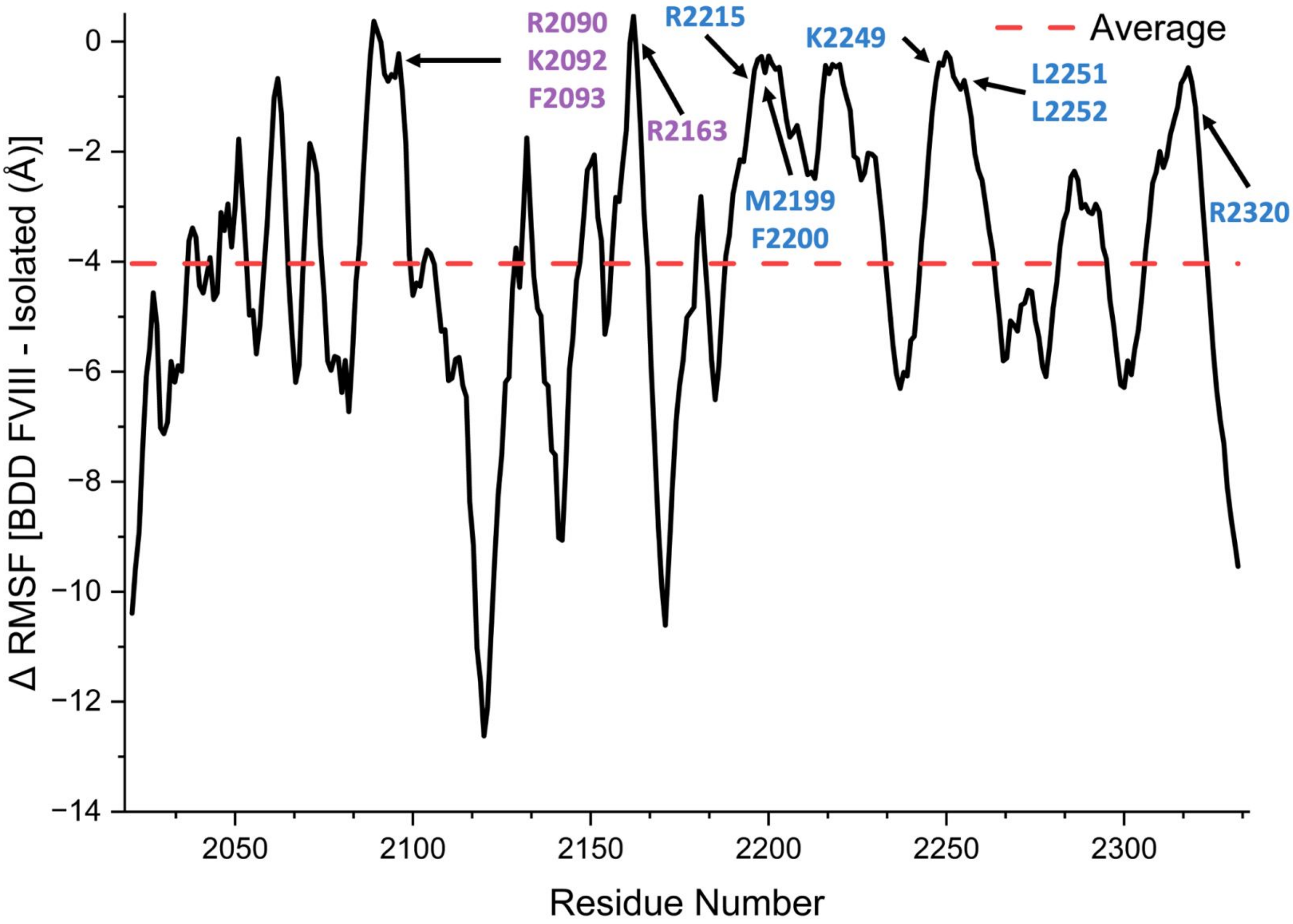

Figure S11. Change in RMSF calculation between BDD FVIII C1 and C2 domains and isolated C1 and C2 domains with a lipid nanodisc simulations. C1 residues are represented in purple and C2 in blue. The average change in RMSF is represented as a red dashed line. Data is representative of two independent trials.

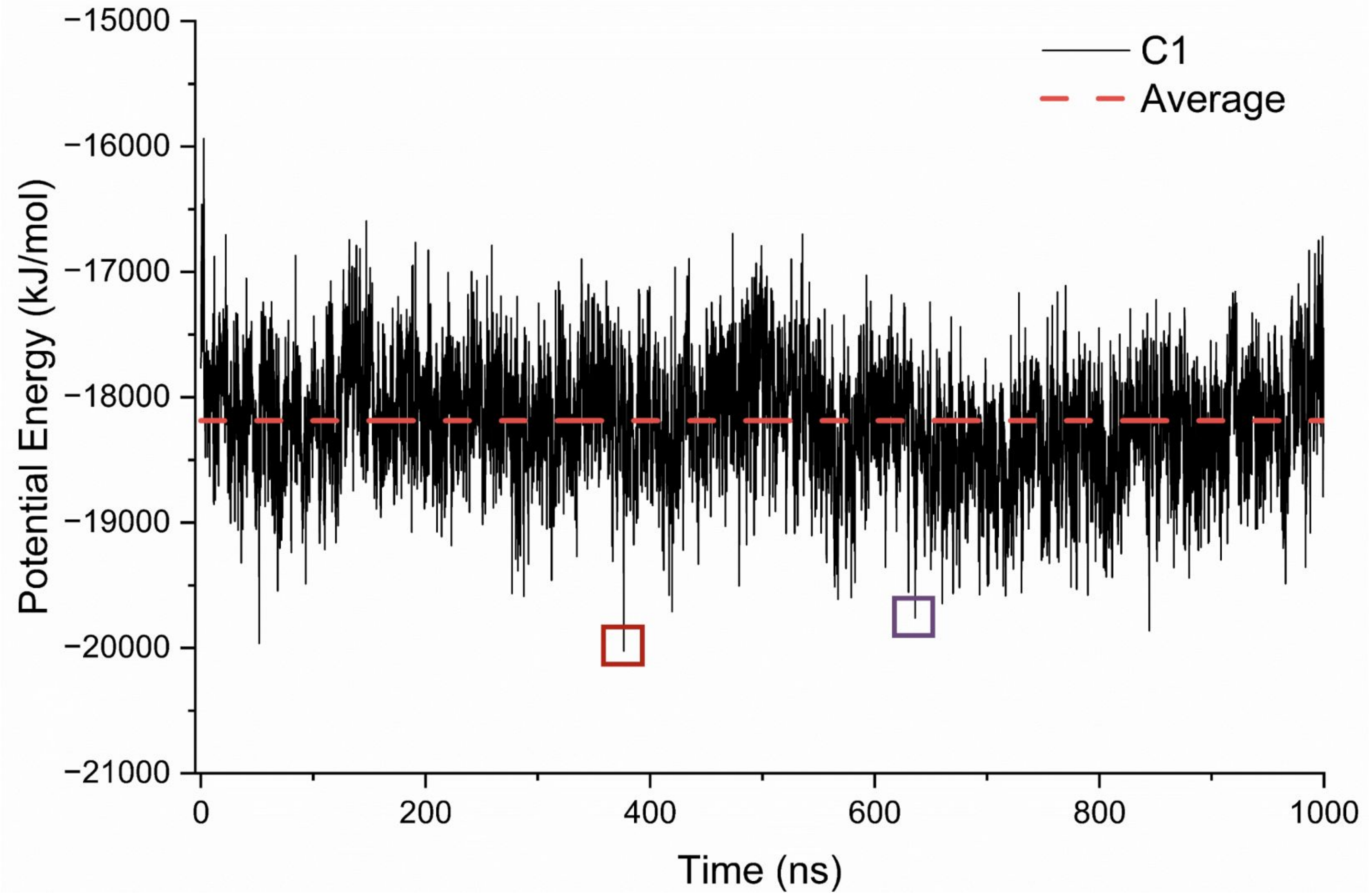

Figure S12. Potential energy between the C1 domain in the isolated C1-nanodisc simulation with DOPC and DOPS. Average potential energy is represented as a red dashed line. The lowest potential energy frame, 1569 (376.560 ns), is boxed in red. The lowest potential energy frame, 2650 (636.000 ns), with R2163 bound to DOPS is boxed in purple. Data are representative of the average of two trials.

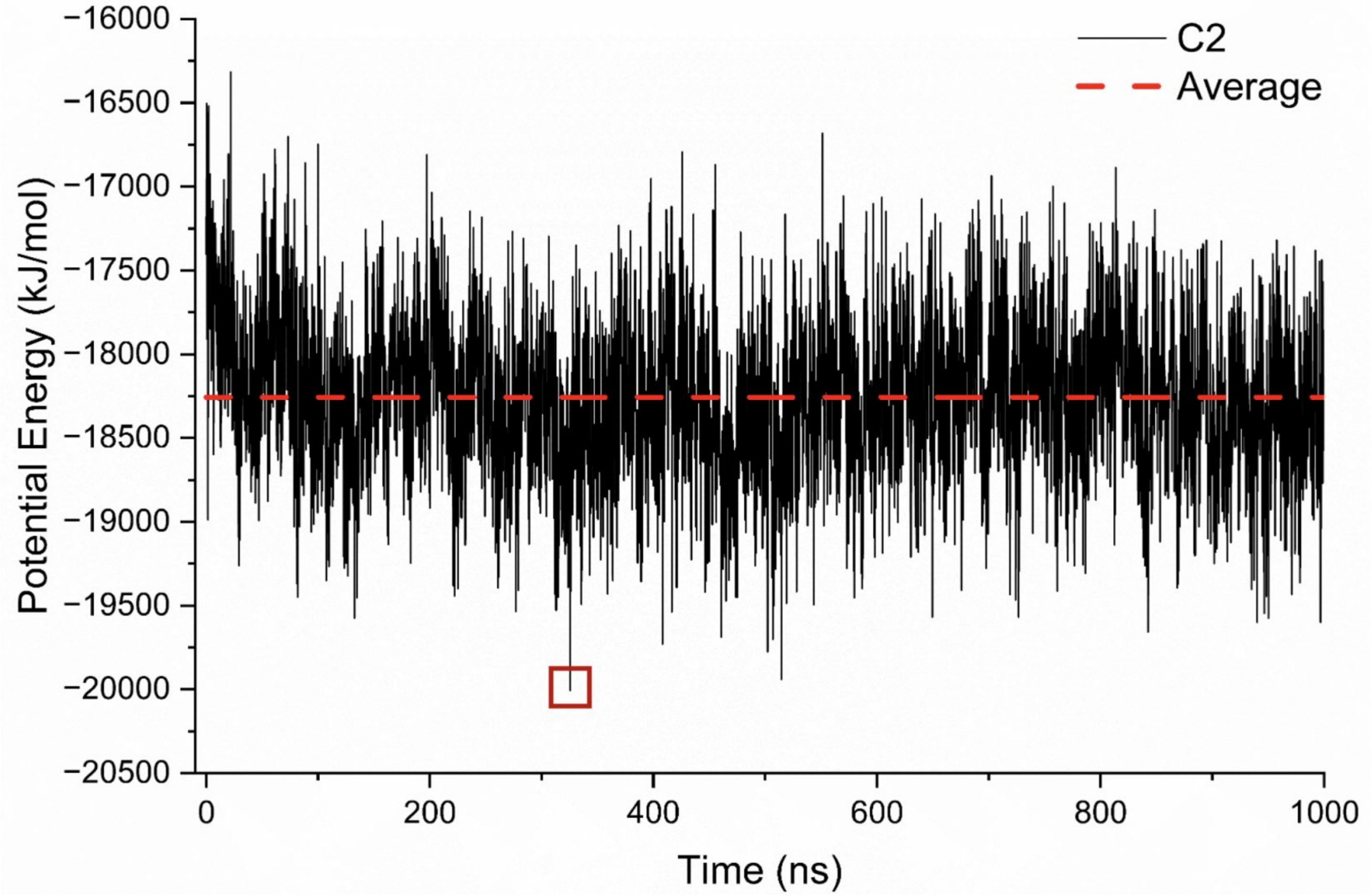

Figure S13. Potential energy between the C2 domain in the isolated C2-nanodisc simulation with DOPC and DOPS. Average potential energy is represented as a red dashed line. The lowest potential energy frame, 1357 (325.680 ns), is boxed in red. Data are representative of the average of two trials.

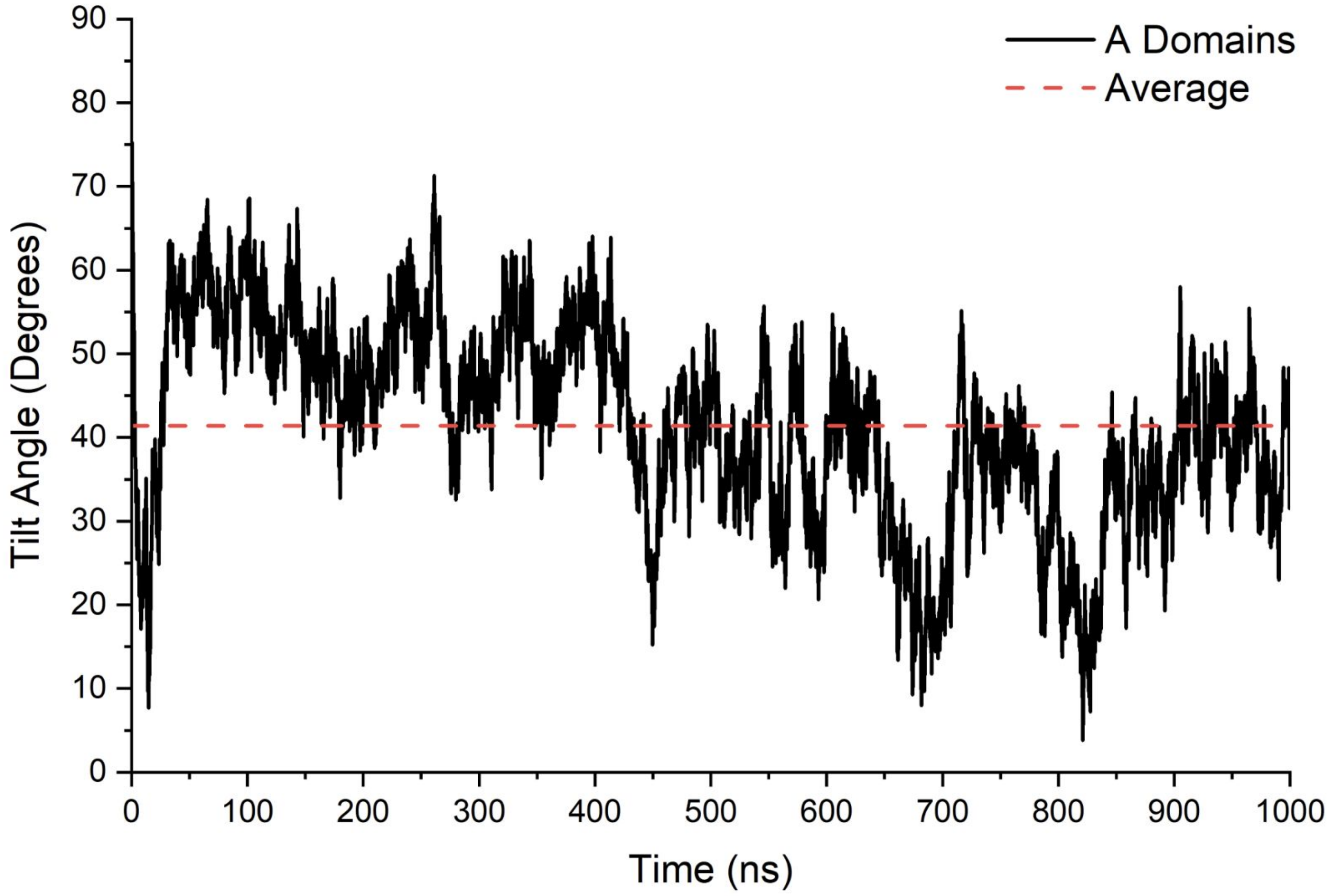

Figure S14. FVIII A domains tilt angle relative to the nanodisc membrane in the FVIII-nanodisc simulations.

The average tilt angle is represented as a red dashed line. Data are representative of the average of two trials.

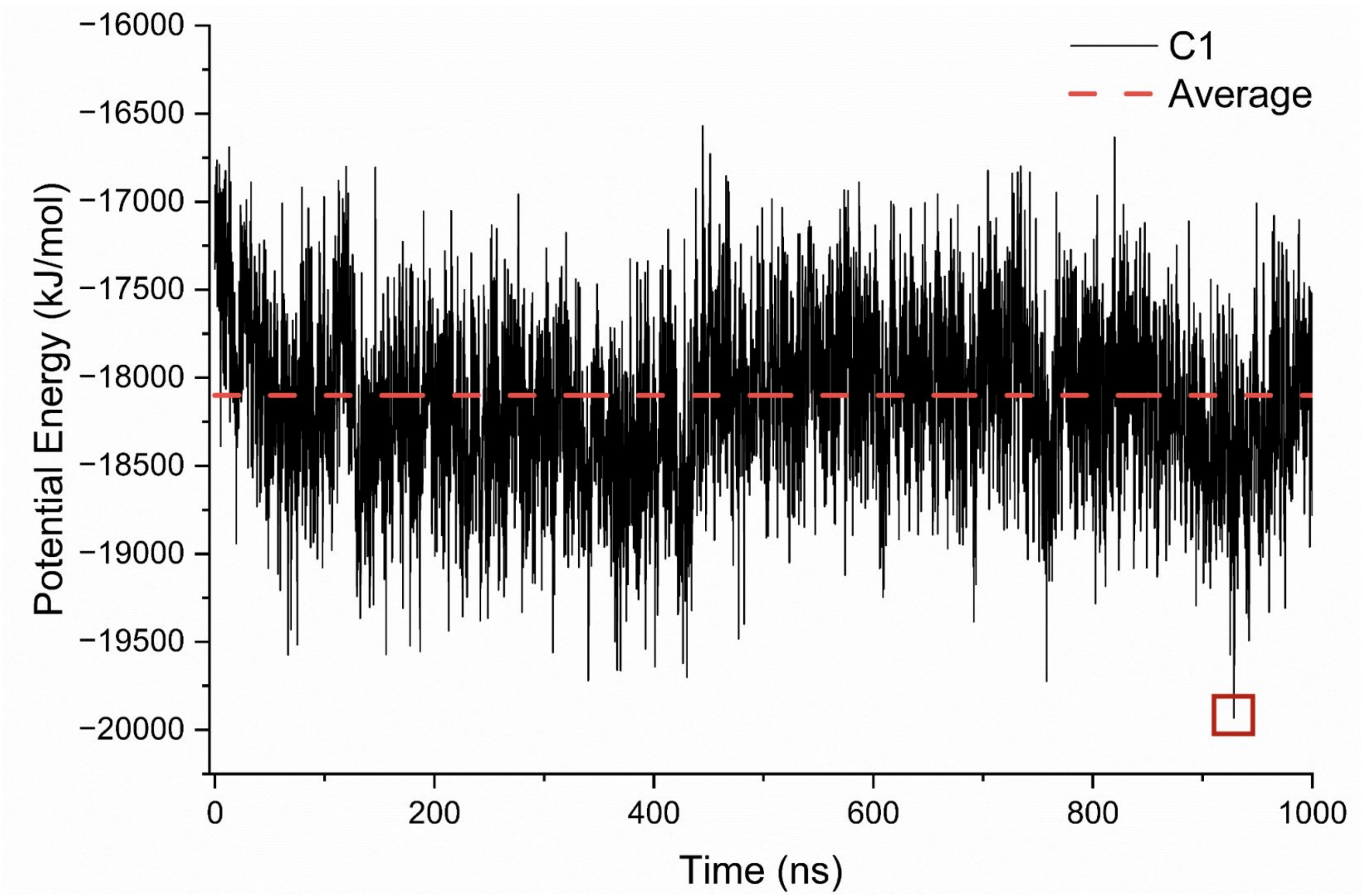

Figure S15. Potential energy between the C1 domain in the BDD FVIII-nanodisc simulations with DOPC and DOPS. The lowest potential energy frame, frame 3,869 (928.560 ns), is boxed in red. Average potential energy is represented as a red dashed line. Data are representative of the average of two trials.

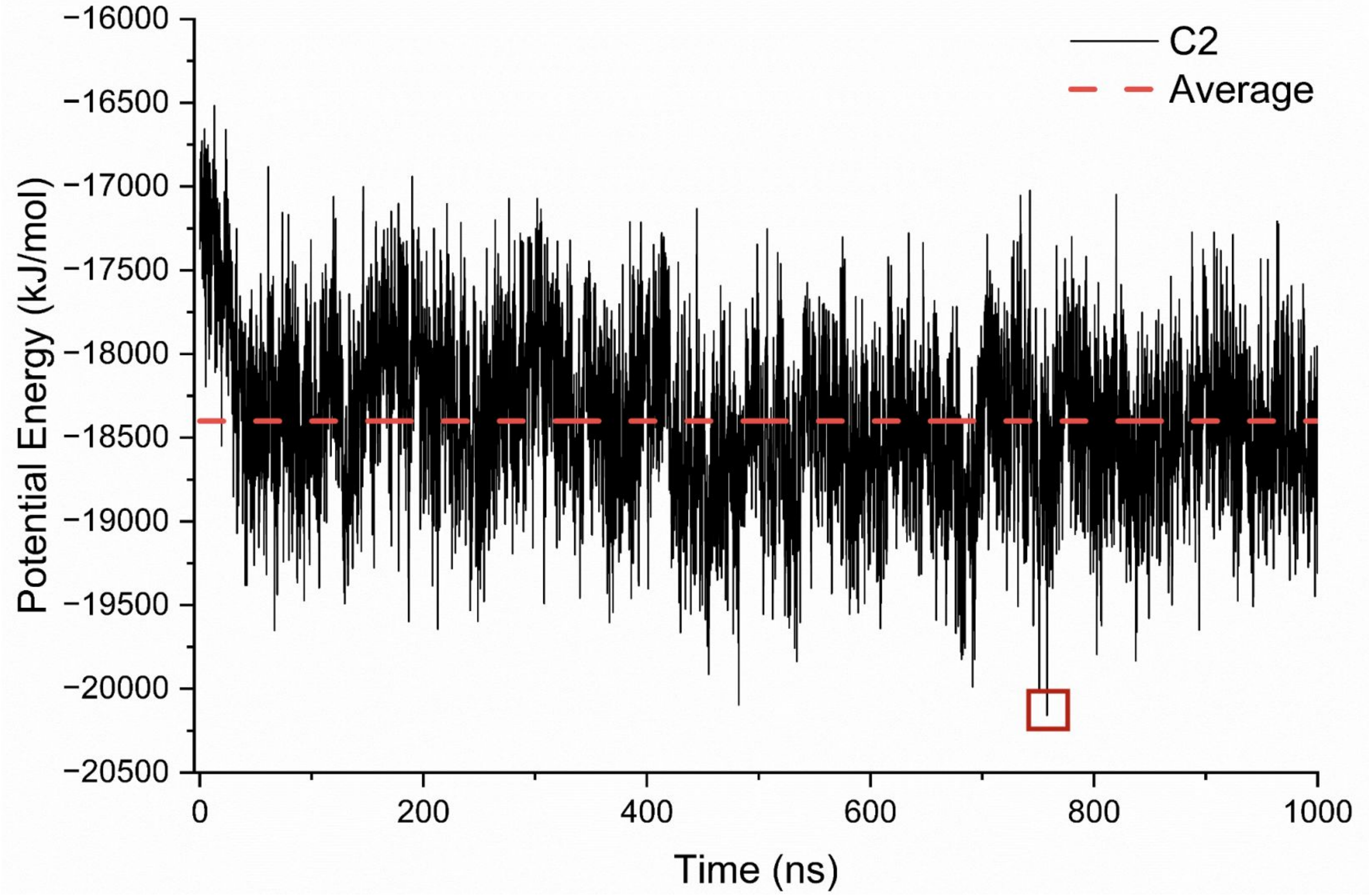

Figure S16. Potential energy between the C2 domain in the BDD FVIII-nanodisc simulations with DOPC and DOPS. Average potential energy is represented as a red dashed line. The lowest potential energy frame, 3158 (757.920 ns), is boxed in red. Data are representative of the average of two trials.

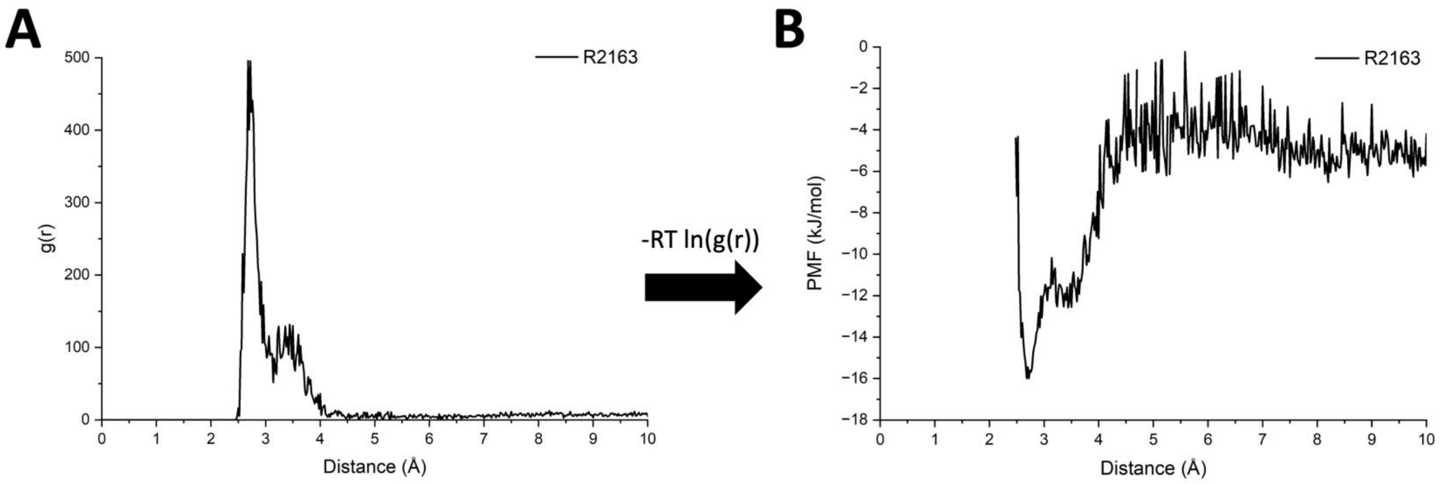

Figure S17. Radial distribution (A) and PMF (B) of guanidino group R2163 (C1) and carboxyl group of DOPS in the isolated C1-nanodisc simulations. Data are representative of the average of two trials.

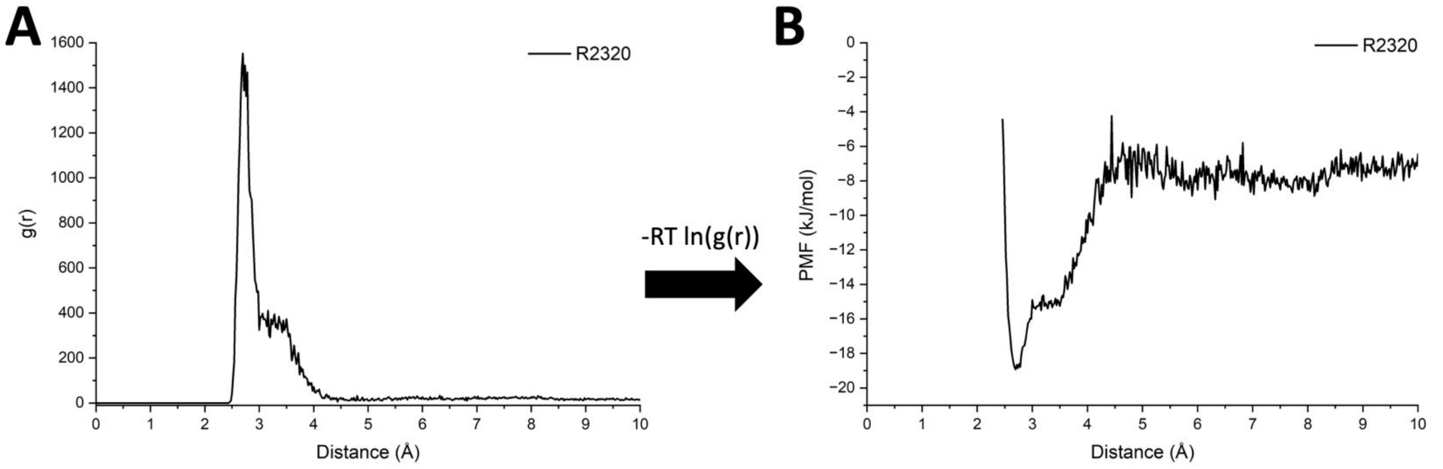

Figure S18. Radial distribution (A) and PMF (B) of guanidino group R2320 (C2) and carboxyl group of DOPS in the isolated C2-nanodisc simulations. Data are representative of the average of two trials.

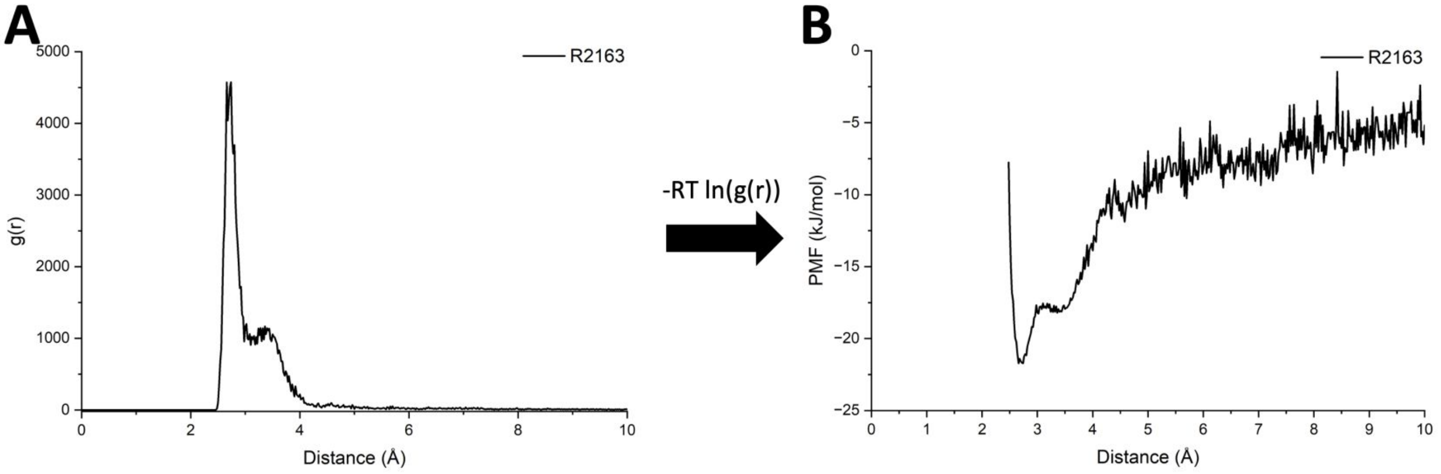

Figure S19. Radial distribution (A) and PMF (B) of guanidino group R2163 (C2) and carboxyl group of DOPS in the BDD FVIII-nanodisc simulations. Data are representative of the average of two trials.

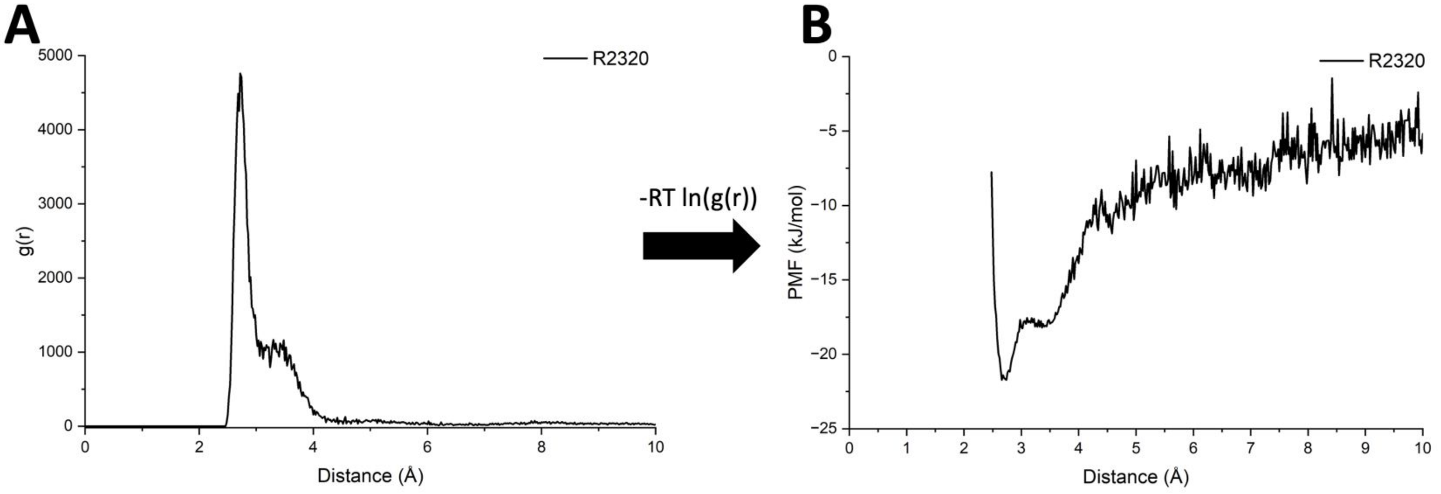

Figure S20. Radial distribution (A) and PMF (B) of guanidino group of R2320 (C2) and carboxyl group of DOPS in the BDD FVIII-nanodisc simulations. Data are representative of the average of two trials.

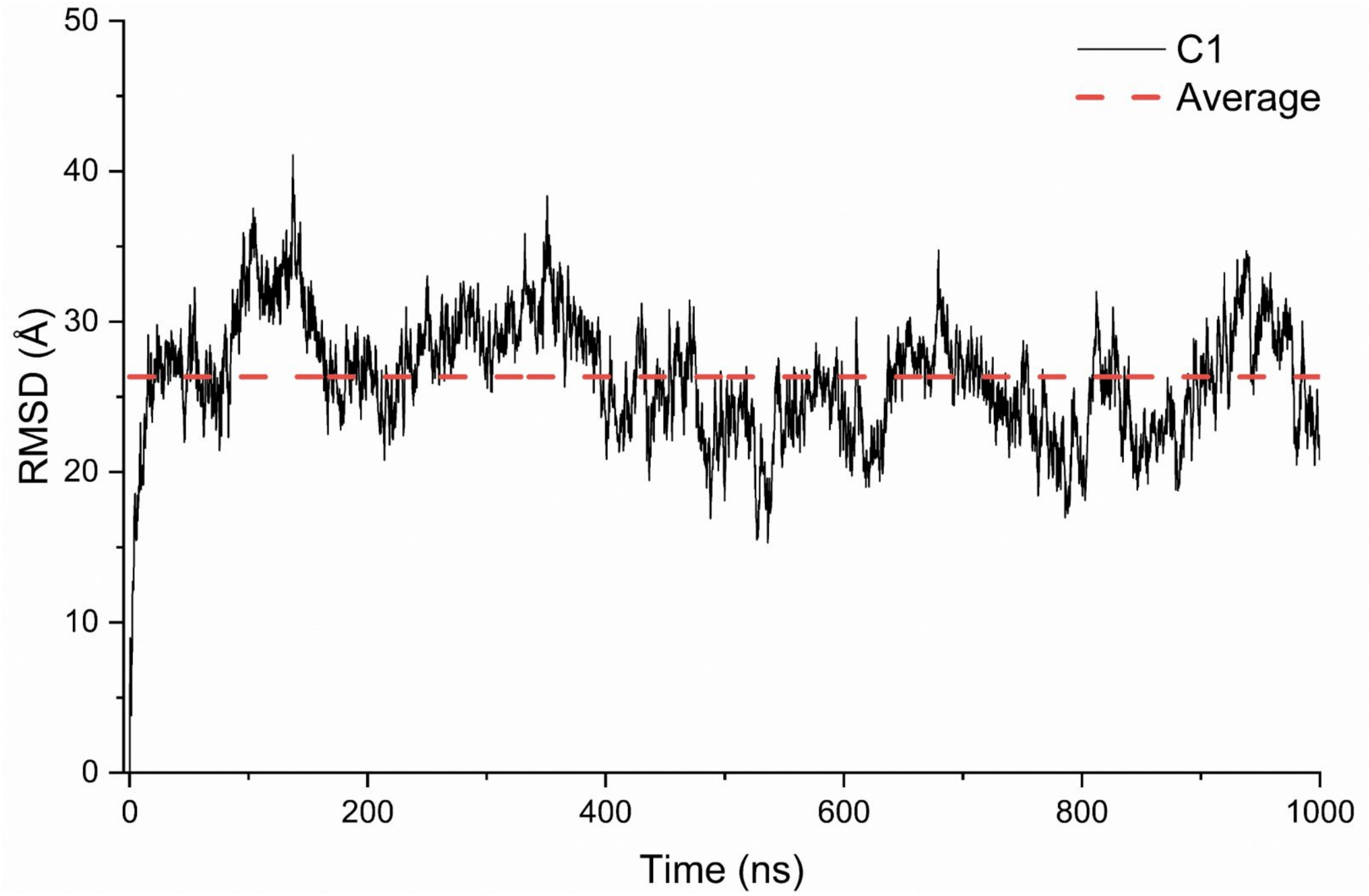

Figure S21. Backbone RMSD of the C1 domain in the isolated C1-nanodisc simulation. Average RMSD is represented as a red dashed line. Data are representative of the average of two trials.

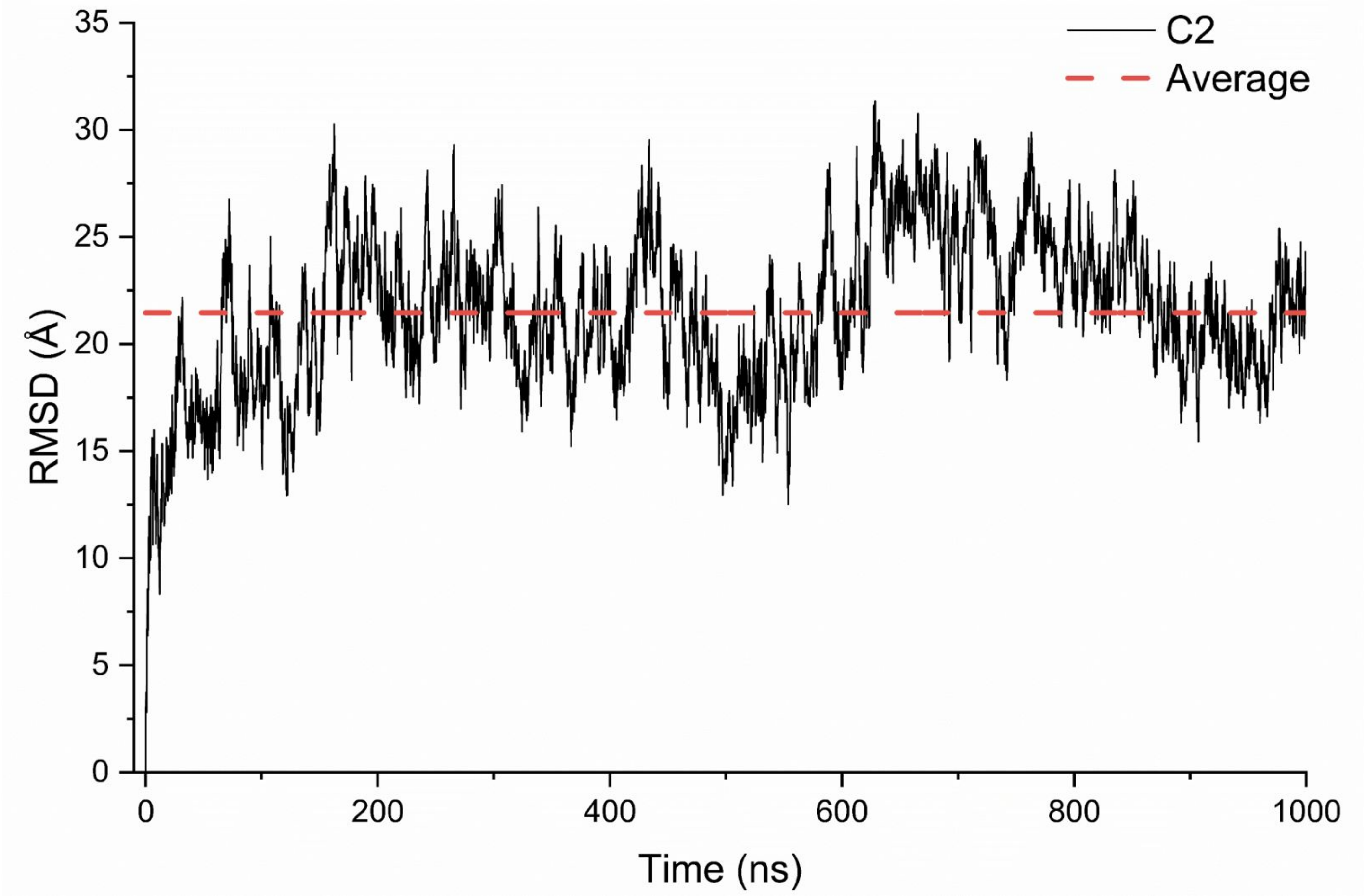

Figure S22. Backbone RMSD of the C2 domain in the isolated C2-nanodisc simulation. Average RMSD is represented as a red dashed line. Data are representative of the average of two trials.

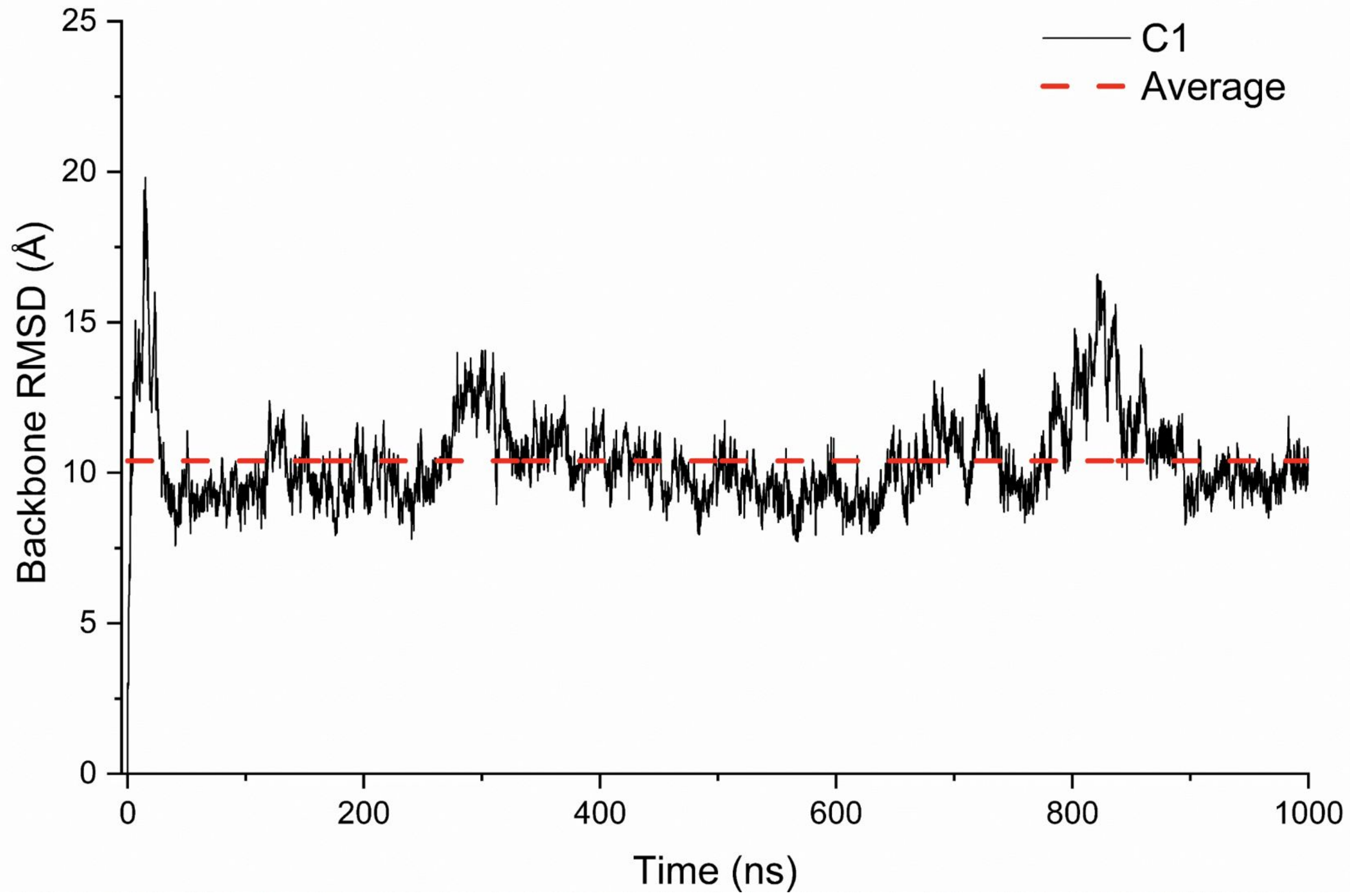

Figure S23. Backbone RMSD of the C1 domain in the BDD FVIII-nanodisc simulation. Average RMSD is represented as a red dashed line. Data are representative of the average of two trials.

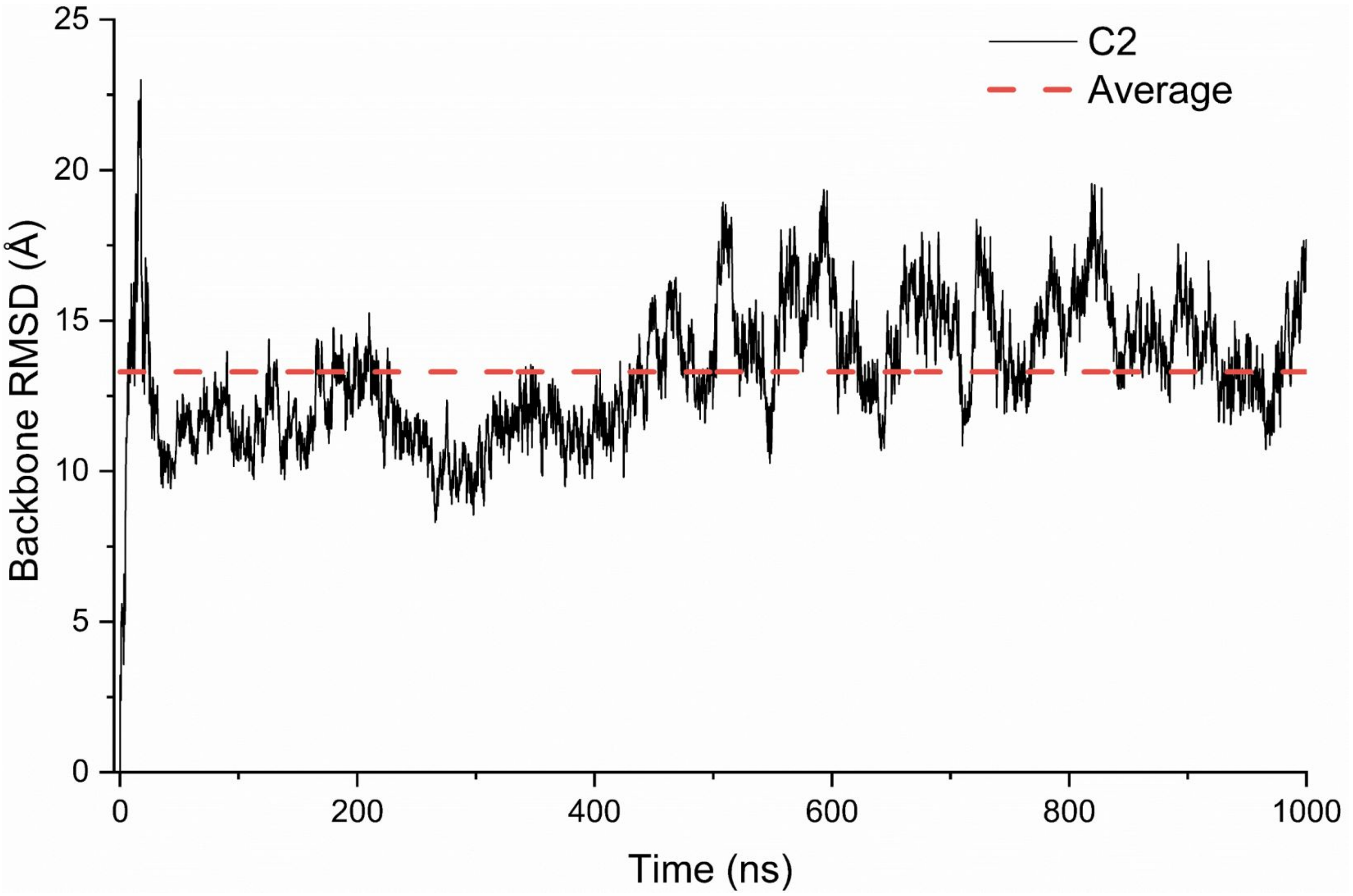

Figure S24. Backbone RMSD of the C2 domain in the BDD FVIII-nanodisc simulation. Average RMSD is represented as a red dashed line. Data are representative of the average of two trials.

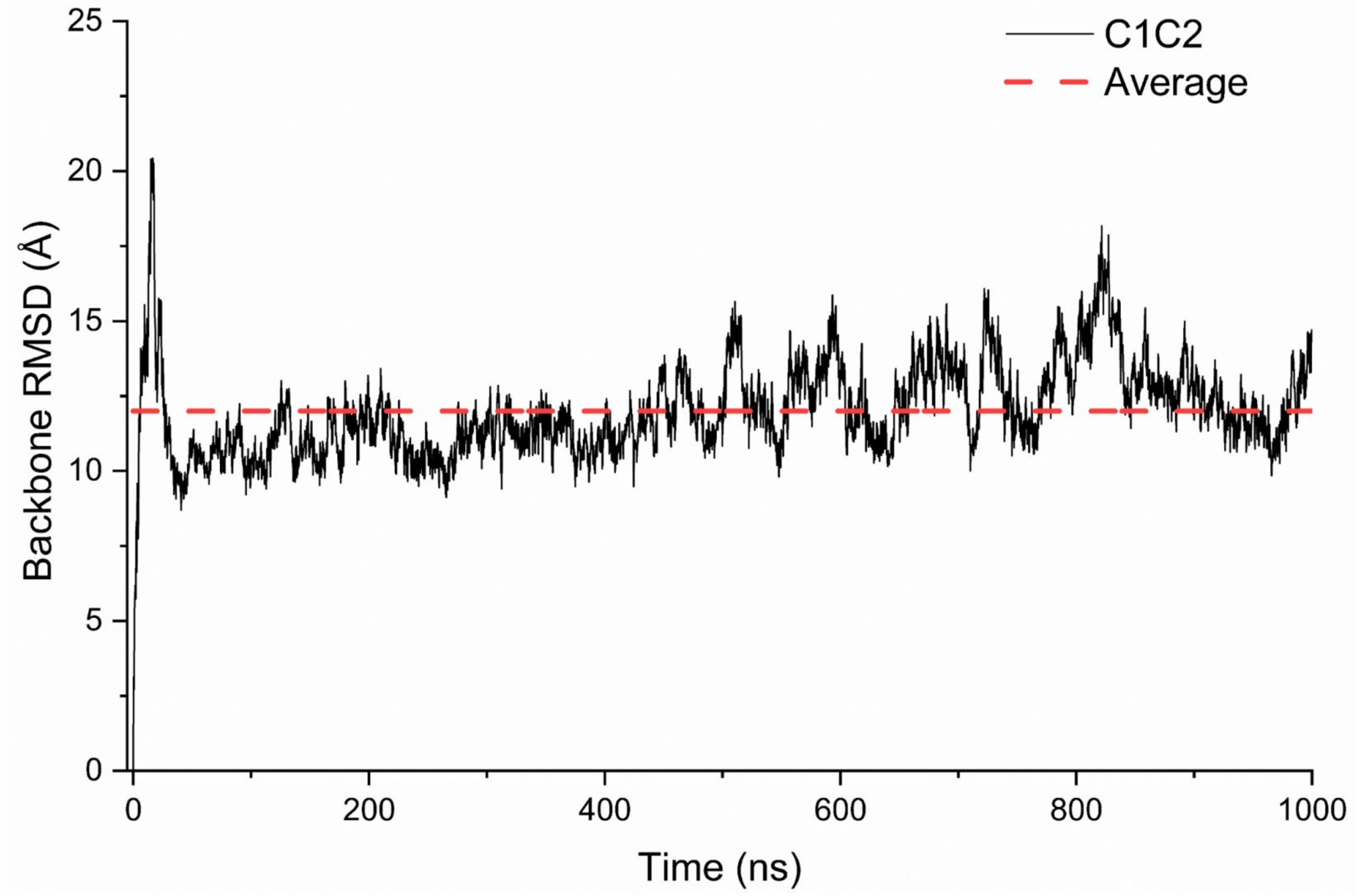

Figure S25. Backbone RMSD of the C1 and C2 domains in the BDD FVIII-nanodisc simulation. Average RMSD is represented as a red dashed line. Data are representative of the average of two trials.
